# Global carbon dioxide removal rates from forest landscape restoration activities

**DOI:** 10.1186/s13021-018-0110-8

**Published:** 2018-11-20

**Authors:** Blanca Bernal, Lara T. Murray, Timothy R. H. Pearson

**Affiliations:** Winrock International, 2121 Crystal Drive Suite 500, Arlington, VA 22202 USA

**Keywords:** Carbon sequestration, Removal factors, Reforestation/afforestation, Plantations, Natural forest regeneration, Agroforestry, Mangrove restoration

## Abstract

**Background:**

Forest landscape restoration (FLR) has been adopted by governments and practitioners across the globe to mitigate and adapt to climate change and restore ecological functions across degraded landscapes. However, the extent to which these activities capture CO_2_ with associated climate mitigation impacts are poorly known, especially in geographies where data on biomass growth of restored forests are limited or do not exist. To fill this gap, we developed biomass accumulation rates for a set of FLR activities (natural regeneration, planted forests and woodlots, agroforestry, and mangrove restoration) across the globe and global CO_2_ removal rates with corresponding confidence intervals, grouped by FLR activity and region/climate.

**Results:**

Planted forests and woodlots were found to have the highest CO_2_ removal rates, ranging from 4.5 to 40.7 t CO^2^ ha^−1^ year^−1^ during the first 20 years of growth. Mangrove tree restoration was the second most efficient FLR at removing CO_2_, with growth rates up to 23.1 t CO_2_ ha^−1^ year^−1^ the first 20 years post restoration. Natural regeneration removal rates were 9.1–18.8 t CO_2_ ha^−1^ year^−1^ during the first 20 years of forest regeneration, followed by agroforestry, the FLR category with the lowest and regionally broad removal rates (10.8–15.6 t CO_2_ ha^−1^ year^−1^). Biomass growth data was most abundant and widely distributed across the world for planted forests and natural regeneration, representing 45% and 32% of all the data points assessed, respectively. Agroforestry studies, were only found in Africa, Asia, and the Latin America and Caribbean regions.

**Conclusion:**

This study represents the most comprehensive review of published literature on tree growth and CO_2_ removals to date, which we operationalized by constructing removal rates for specific FLR activities across the globe. These rates can easily be applied by practitioners and decision-makers seeking to better understand the positive climate mitigation impacts of existing or planned FLR actions, or by countries making restoration pledges under the Bonn Challenge Commitments or fulfilling Nationally Determined Contributions to the UNFCCC, thereby helping boost FLR efforts world-wide.

**Electronic supplementary material:**

The online version of this article (10.1186/s13021-018-0110-8) contains supplementary material, which is available to authorized users.

## Background

Global emissions from deforestation and forest degradation have been historically high [1–5]. Hansen et al. [6] estimated a gross forest loss of 2.3 million km^2^ worldwide between 2000 and 2012, while net deforestation between 2010 and 2015 is reported to be 1.3 million km^2^ [7, 8]. Most deforestation and forest degradation is concentrated in the tropics, a region responsible of 3.7 Gt CO_2_ year^−1^ emissions from deforestation between 2000 and 2012 [9], averaging 6.2 Gt CO_2_ year^−1^ between 2005 and 2010 [4]. Emissions from forest degradation due to fires, timber harvest, and fuelwood yielded a further 2.1 Gt CO_2_ year^−1^ during that same period [4]. Forest loss and degradation not only entail emissions from carbon stored in biomass, but also the loss of a continuous atmospheric CO_2_ sink, threatening our ability to abate increasing greenhouse gas (GHG) emissions to the atmosphere and to mitigate climate change.

The negative effects of these emissions on global climate are compounded by the loss of ecosystem services associated with the decrease in forest habitat [10, 11]. The already-felt impacts of these losses, paired with continued diminishment of forests and ecosystem services, has brought global interest in conserving remaining forests and restoring those previously damaged and lost. The international community thereby seeks both to mitigate losses and to promote sustainable use of forests in the face of a growing global population and increasing demand for land and resources [11–13].

Accordingly, international restoration programs have been growing, engaging country governments, private sector entities, and civil society organizations to re-establish tree cover across landscapes [14, 15]. Most notably, under the Bonn Challenge, over 47 countries have committed to restore 150 million hectares by 2020 and 350 million hectares by 2030. The Bonn Challenge was endorsed and extended in 2014 by the New York Declaration on Forests [16], pledging to cut 16.5–32.3 Gt CO_2_ annual emissions from natural forest loss. While many countries have included restoration activities as part of their Nationally Determined Contributions (NDCs) towards the 2014 UNFCCC Paris Agreement and in their strategies to reduce emissions from deforestation and forest degradation (REDD+), very few include a CO_2_ removal target through forest restoration in their NDCs, and even fewer have these quantitative forest restoration targets aligned with their Bonn Challenge commitments [17]. While forest restoration is a well-known efficient climate mitigation solution [18], the CO_2_ removals these activities can achieve are poorly known in many countries where these data are limited or do not exist and while there is no comprehensive reference of removal values to apply to these actions.

To understand the volume of atmospheric CO_2_ removal benefits from carbon sequestration through forest restoration, the rate of biomass growth (i.e., the increase of tree biomass over time) under a restoration activity needs to be calculated. This rate varies with climate, landscape characteristics, tree species, management practices, and forest restoration approaches [19]. IPCC Guidelines [20] offer broad, continental values termed tier 1 defaults on aboveground net biomass growth for natural forests along with tropical and subtropical forest plantations, across geophysical categories and for common planted tree species. However, the massive regions these defaults apply to can lead to great divergence from reality and thus low credibility for resulting numbers, for which the IPCC does not provide standard statistical information to assess their accuracy [21, 22]. Furthermore, many forest restoration activities are not covered at all by these generic defaults [19]. Langner et al. [21] determined that when the IPCC defaults are used for non-intact tropical forests, their overestimation of biomass growth could be up to 35% of what pan-tropical biomass maps [23, 24] predict.

Under REDD+, and arguably under any restoration program, growth rates for specific restoration types and locations are needed and should be more accurate than the available tier 1 defaults. Attaining the appropriate growth rates, however, relies on the availability of data derived from studies assessing biomass growth in the geography and under conditions that adequately match the forest restoration activity undertaken. This type of data across regions and restoration activities is often unavailable, as it requires consistent monitoring of biomass growth over years or decades, making this type of research resource intensive.

Current datasets available do not fulfill the need to assess the potential CO_2_ removals of different types of restoration actions worldwide. While some tools exist for quantifying potential impact from specific tree planting activities using models (e.g., the US Forest Service Carbon Online Estimator [25], the Forest Vegetation Simulator [26], COMET-VR [27], and Global Forest Biodiversity Initiative Forest Inventory [28, 29]), they are specific to limited geographies or tree planting activities. Large-scale evaluations of published data on tree growth focus on evaluating the influence particular variables have on the success of afforestation activities [30–35], relevant for elucidating improved techniques and approaches for ecological restoration. Yet these studies fail to offer standard rates that could be used by practitioners to estimate the climate impact of forest landscape restoration (hereafter *FLR*) across different latitudes.

To fill this gap, we conducted a literature review of biomass accumulation rates from FLR activities across the globe, using published and scientifically-validated data, and developed CO_2_ removals rates (i.e., removal factors) for four FLR categories that we used to build a Global CO_2_ Removals Database (publicly available for download [36]) that indicates what removal rates apply to each subnational unit in the world. Our Database is a resource to practitioners and officials looking to evaluate the impact of past or future FLR activities at the national or subnational level, especially where FLR category- or geographically-specific removals rates are not available. This paper expands on the information provided in the Global CO_2_ Removals Database, detailing the development of the CO_2_ removal rates for four distinct FLR activities in regions and climates across the globe and their corresponding uncertainty (not included in the Database), summarizing the main findings of this analysis, their implications, and highlighting remaining gaps. The removal rates from biomass accumulation in FLR developed in this study can facilitate decision-making by increasing understanding of potential FLR climate mitigation benefits and providing a credible set of annual CO_2_ removal estimates to guide practitioners and policy makers when assessing the CO_2_ removal potential of different forest restoration options.

## Methods

FLR encompasses a wide array of activities that have been categorized in the Restoration Opportunities Assessment Methodology, ROAM [19]. These activities can take place in (i) forest land if forests are the main land use (e.g., planted forests and woodlots or natural regeneration in deforested land, or silviculture in degraded forest land); (ii) agricultural land if land is managed for food production (e.g., agroforestry in permanently managed lands, or improved fallow in those sporadically managed); and (iii) protective lands or buffers if the land is either threatened by changes in the environment and climate or key in protecting communities from said changes (e.g., mangrove restoration in coastlines, or watershed protection and erosion control). Of all these FLR activities, we studied the biomass growth and CO_2_ sequestration of planted forests and woodlots, natural regeneration, agroforestry, and mangrove restoration. We did not include silviculture, improved fallow, or watershed protection because of the wide range of activities they encompass and the unspecific correlation between each of them and the quantitative biomass increase they would yield in the landscape. Specific assumptions made under each of these FLR activities for the compilation of this global database, as well as the methodology followed to develop removal factors, are described below.

### Review of FLR data

**Fig. 1:**
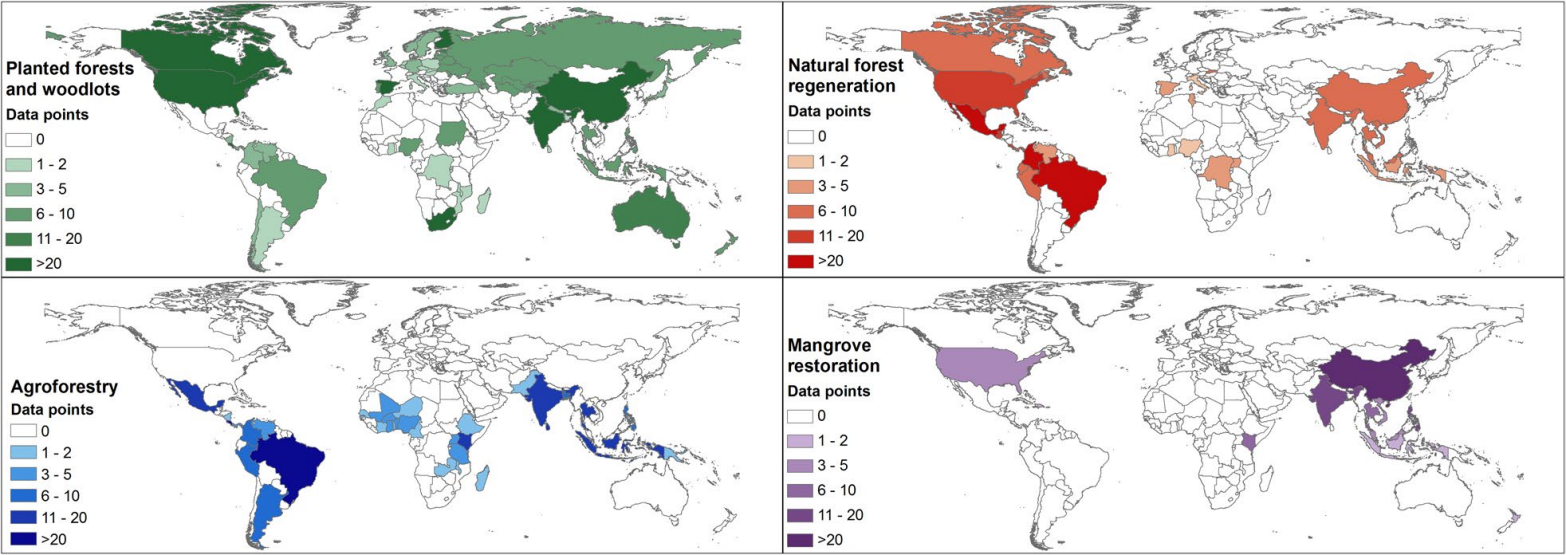
Regional distribution of data points collected on each of the FLR activities (planted forests and woodlots, natural forest regeneration, agroforestry, and mangrove restoration) from peer-reviewed manuscripts and published reports. Data points represent stands of known age and aboveground biomass stock (tons ha^−1^)

We reviewed over 335 scientific peer-reviewed manuscripts and published reports (Additional file 1) that yielded 1197 independent data points on plot aboveground biomass carbon pools associated with a stand age, reflecting the four selected FLR activities: planted forests and woodlots, natural regeneration, agroforestry, and mangrove restoration (Fig. 1). The data collected therefore represents common practices implemented in these four FLR activities.

Where reported aboveground biomass stocks were expressed in units other than tons of carbon per hectare, they were converted as follows: (i) if the reported units were mass of biomass per area, they were converted to mass carbon per area by applying the carbon fraction of dry matter conversion factor of 0.47 (per IPCC Guidelines [20]); or (ii) if the reported units were mean annual volume increment (MAI), they were converted to carbon biomass by multiplying the MAI by the reported stand age and by the planted species wood density (obtained from IPCC Guidelines [20] and Zanne et al. [37]) to calculate biomass per area, which was then converted to mass of carbon per area.

Geographical locations of data points (coordinates or location of the data points within a country) were mapped against the global Köppen–Geiger Climate Classification [38] to determine the climate of the FLR sites. Broadly, this classification defines the climate as boreal, temperate, tropical, and subtropical according to latitude and altitude, and as dry and humid forest types according to annual precipitation.

### Planted forests and woodlots

The planted forests and woodlots FLR category covered plantation monocultures, typically on previously cleared land. In cases where comparative studies reported biomass under control (unaltered) and experimental conditions, only the data from the control parcels were used. To decrease data variability, plantation data were grouped by planted species. The species categories were: teak (*Tectona grandis*), eucalyptus (*Eucalyptus* sp.), pine (*Pinus* sp.), oak (*Quercus* sp.), generic broadleaf excluding teak and eucalyptus (e.g., *Populus* sp., *Gmelina* sp., or *Leucaena* sp., among others), and generic conifer excluding pine (e.g., *Cupressus* sp., *Abies* sp., etc.). Data within these species groupings were further subdivided by climate and forest type. We did not include removal factors of planted forests and woodlots for stands older than 20 years in this study based on the assumption that most planted forests and woodlots are felled before that age. Data from tropical climates under this FLR category represents data from both tropical and subtropical regions.

### Natural regeneration

Successional forests, secondary forests, and forest restoration activities not characterized as enrichment planting are included under the natural regeneration FLR category. Forest restoration activities entailing silvicultural or management practices are therefore excluded. Naturally regenerated forests are typically dominated by a diverse mix of species and therefore were grouped by region (Asia and Oceania, Europe, Africa, North America, Central America, and South America) rather than species type. Each category was further subdivided into dry and humid forest types.

### Agroforestry

Agroforestry FLR encompasses all activities that combine trees with an agricultural landscape (i.e., crops or livestock). These include multistrata, tree intercropping, silvopastoral, and protective systems, reflecting a wide range of CO_2_ sequestration potential. Available data on agroforestry CO_2_ sequestration allowed for regional categorization (Latin America and Caribbean, Africa, and Asia), but not by agroforestry type. A lack of enough data publicly available on plot biomass stock per agroforestry stand age for these agroforestry types in Europe and North America prevented the development of robust agroforestry growth curves for these regions under this study.

### Mangrove restoration

This FLR category included only mangrove sites in the tropics and subtropics where restoration was achieved by actively planting mangroves, rather than by restoring hydrological conditions and allowing the mangroves to naturally colonize the site. To reduce data variability, mangroves were grouped as tree and shrub based on the stand description, climatic region (tropical and subtropical), and species [39, 40].

### Development of removal factors

We developed specific biomass growth curves for each subcategory of the selected FLR activities (Additional files 1, 2), based on the Chapman-Richards equation [41, 42]. These growth curves plot net cumulative aboveground carbon stocks (tons C ha^−1^) against stand age as a sigmoid function that can be used to estimate past and future biomass growth. Following IPCC Guidelines [20], we estimated pre- and post-20-year growth rates (tons C ha^−1^ year^−1^) of each FLR subcategory, as well as its precision (95% confidence interval of the estimate). Pre-20-year rates (0–20 years of growth) were calculated as the cumulative aboveground biomass pool at year 20 divided by 20 years; post-20-year rates (20–60 years of growth) were calculated as the cumulative aboveground biomass pool at age 60 minus that at age 20, and the result was then divided by 40 years. Dividing the cumulative biomass growth of a stand by its age provides the simplifying assumptions of a constant growth rate over the selected period [20].

We calculated pre- and post-20-year belowground biomass growth corresponding to each of the FLR subcategories following Mokany et al. [43], whereby belowground biomass is estimated as a function of the calculated aboveground biomass. The sum of the aboveground and belowground biomass growth rates are then presented as total tons C ha^−1^ year^−1^ captured by each FLR activity in each climate and region, then converted to annual removal factors as carbon dioxide (tons CO_2_ ha^−1^ year^−1^) by multiplying the tons of C by the factor 3.66 (i.e., 44 g CO_2_ over 12 g C).

We calculated the 95% confidence intervals (hereafter *CI95*) of the Chapman-Richards aboveground biomass growth curves. To estimate the CI95 of our belowground biomass calculations, we computed the percentage uncertainty of the root:shoot ratios in Mokany et al. [43] for each of their “vegetation categories” (i.e., forest climates and regions) by calculating the CI95 of these ratios and dividing it by their median. The percentage uncertainty was then applied to our belowground biomass growth rates to estimate their CI95. Through the error propagation of the sum of aboveground and belowground CI95s [20], we estimated the CI95 of the total growth rates. We did not evaluate the uncertainty of the allometric models or other default factors in the literature datasets and thus, the estimates of uncertainty reported in this study capture the prevalence and variability in the input data. Lastly, we explored the relationship between planted species and climate with a linear regression (95% confidence interval) and determined the differences between them with a single factor ANOVA (*α* = 0.05).

## Results

**Fig. 2:**
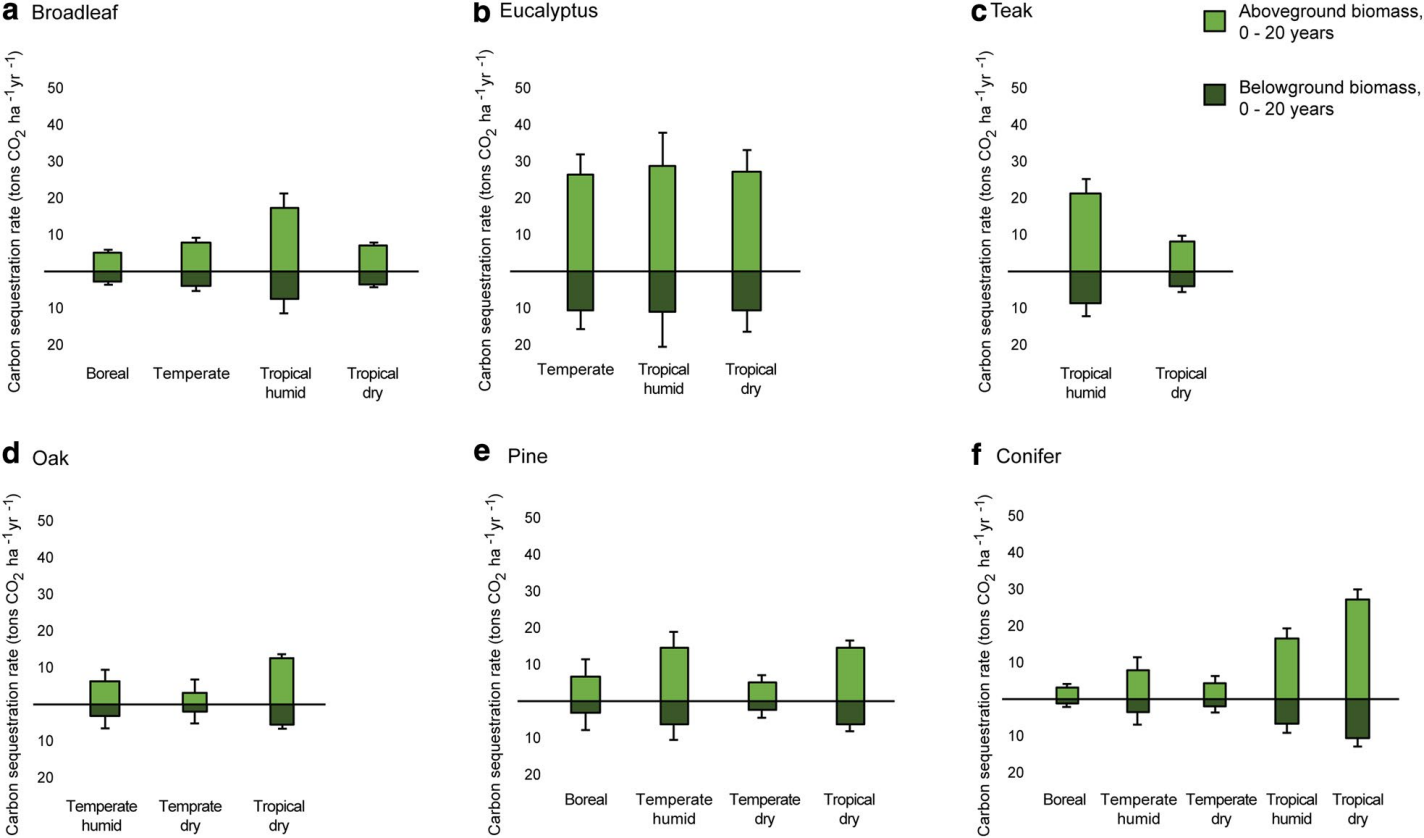
Carbon sequestration rate (tons CO_2_ ha^−1^ year^−1^) of the six plantations and woodlots groups (**a** broadleaf excluding eucalyptus and teak, **b** eucalyptus, **c** teak, **d** oak, **e** pine, and **f** conifers excluding pine) during the first 20 years of tree growth. Light green represents aboveground biomass, while dark green represents belowground biomass. Error bars indicate the CI95 of the total biomass growth. Different bars within graphs represent climatic regions

Removal factors for the first 20 years of growth ranged from 4.5 to 40.7 t CO_2_ ha^−1^ year^−1^ (Tables 1, 2). Planted forests and woodlots, typically managed to maximize growth, presented the highest removal factors. Among the species under the planted forests and woodlots FLR category, those in tropical regions showed the highest removal factors (e.g., conifers, oak, or broadleaf species), while eucalyptus remained high across climates (Fig. 2). Broadleaves and conifers, including pine, had the widest latitudinal range, serving as the only species with sufficient data in boreal climates. Growth data on oaks and eucalyptus were only available for temperate and tropical regions, while teak was limited to the tropics. The planted forests and woodlots FLR category benefitted from the greatest data abundance, yet two relevant limitations were identified: (i) data were scarce on temperate dry oaks and tropical dry conifers and broadleaves (*n* = 6; Table 2); and (ii) teak data was abundant yet highly variable (yielding low *R*^2^ in both tropical climates; Table 3) even though tropical humid eucalyptus had the widest 95% confidence interval (Fig. 2) of all planted forests and woodlots subcategories.

**Table 1:** Removal factors (tons CO_2_ ha^−1^ year^−1^) and associated uncertainty (CI95) of the planted forests and woodlots FLR and subcategories, for stand ages of 0–20 years old

Planted species	Climatic region	Removal rate (t CO_2_ ha^−1^ year^−1^)	Half CI95
Oak	Temperate, humid	9.5	3.5
	Temperate, dry	5.3	3.5
	Tropical, dry	18.4	1.0
Teak	Tropical, humid	30.8	4.1
	Tropical, dry	12.7	1.5
Eucalyptus	Temperate, all	37.9	5.5
	Tropical, humid	40.7	9.5
	Tropical, dry	38.8	6.0
Broadleaf^a^	Boreal	8.0	1.0
	Temperate, all	11.8	1.4
	Tropical, humid	25.3	3.9
	Tropical, dry	10.7	0.6
Pine	Boreal	10.2	4.9
	Temperate, humid	21.1	4.5
	Temperate, dry	7.6	2.0
	Tropical, dry	21.0	2.0
Conifer^b^	Boreal	4.5	1.0
	Temperate, humid	11.6	3.6
	Temperate, dry	6.4	1.9
	Tropical, humid	23.6	2.8
	Tropical, dry	38.7	2.5

^a^Excluding eucalyptus and teak

^b^Excluding pine

**Table 2:** Removal factors (tons CO_2_ ha^−1^ year^−1^) and associated uncertainty (CI95) of the natural regeneration, agroforestry, and mangrove restoration FLR activities and subcategories, for stand ages of 0–20 years and 20–60 years

FLR activity	Climatic region	0–20 years		20–60 years	
		Removal rate (t CO_2_ ha^−1^ year^−1^)	Half CI95	Removal rate (t CO_2_ ha^−1^ year^−1^)	Half CI95
Natural regeneration					
Asia and Oceania	Humid	11.9	3.0	17.3	1.2
	Dry	10.3	1.7	3.5	0.9
Europe	All	9.8	1.7	4.5	0.8
Africa	Humid	17.4	2.1	7.9	1.7
North America	Humid	11.1	3.3	10.9	1.8
	Dry	9.1	2.1	8.2	1.2
Central America and Caribbean	Humid	11.9	1.7	7.1	1.5
	Dry	10.4	1.4	0.2	0.7
South America	Humid	18.8	2.0	5.2	1.4
	Dry	13.8	3.3	3.1	1.6
Agroforestry					
Africa	All	10.8	1.7	0.1	0.8
Asia	All	14.0	2.5	0.0	0.4
Latin America and Caribbean	All	15.6	2.7	0.6	0.2
Mangrove restoration					
Tree	Tropical	23.1	2.9	10.7	2.3
Shrub	Tropical and subtropical	6.7	1.5	1.7	0.5

**Table 3:** Coefficient of determination (R^2^) showing the fitness of the growth curves and number of data points (n) used to develop them for each subcategory of the selected FLR categories (i.e., planted forests and woodlots, natural regeneration, agroforestry, and mangrove restoration)

	Climatic region	R^2^	n
Planted forests and woodlots			
Oak	Temperate, humid	0.63	11
	Temperate, dry	0.63	6
	Tropical, dry	0.91	13
Teak	Tropical, humid	0.30	34
	Tropical, dry	0.43	25
Eucalyptus	Temperate, all	0.86	52
	Tropical, humid	0.52	13
	Tropical, dry	0.62	32
Broadleaf	Boreal	0.99	13
	Temperate, all	0.68	77
	Tropical, humid	0.51	54
	Tropical, dry	0.99	6
Pine	Boreal	0.53	10
	Temperate, humid	0.47	41
	Temperate, dry	0.40	12
	Tropical, dry	0.69	28
Conifers	Boreal	0.75	22
	Temperate, humid	0.65	41
	Temperate, dry	0.85	13
	Tropical, humid	0.60	24
	Tropical, dry	0.93	6
Natural regeneration			
Asia and Oceania	Humid	0.58	32
	Dry	0.47	4
Europe	All	0.68	10
Africa	Humid	0.65	8
North America	Humid	0.68	16
	Dry	0.45	53
Central America and Caribbean	Humid	0.65	65
	Dry	0.91	24
South America	Humid	0.31	106
	Dry	0.26	72
Agroforestry			
Africa	All	0.19	52
Asia	All	0.13	77
Latin America and Caribbean	All	0.21	82
Mangrove restoration			
Tree	Tropical	0.57	50
Shrub	Tropical and subtropical	0.53	13

**Fig. 3:**
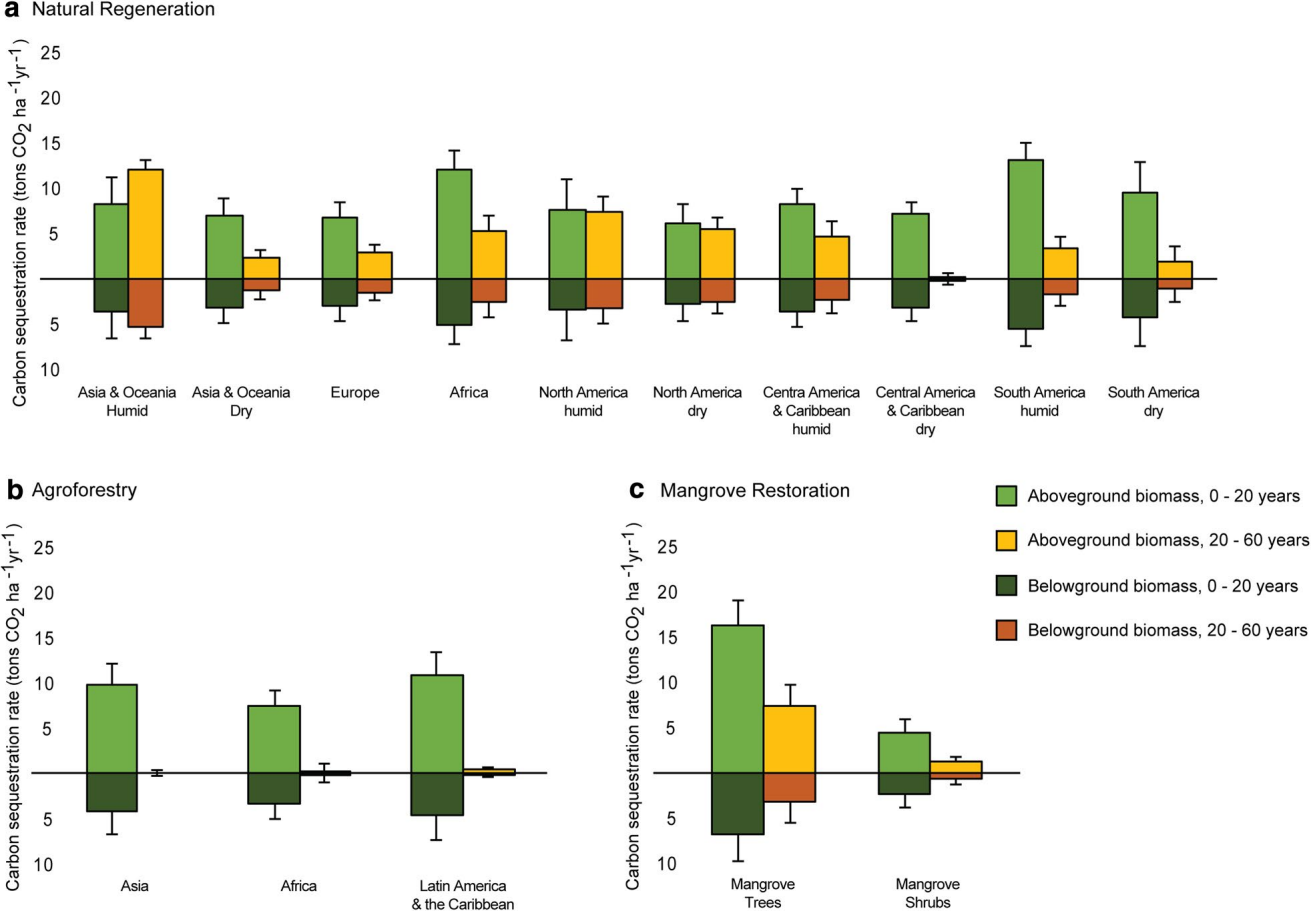
Carbon sequestration rate (tons CO_2_ ha^−1^ year^−1^) of **a** natural regeneration FLR, **b** agroforestry FLR, and **c** mangrove restoration FLR. Green colors represent rates during the first 20 years of tree growth (aboveground biomass in light green, belowground biomass in dark green), while orange colors represent rates during 20–60 years of tree growth (aboveground biomass in light orange, belowground biomass in dark orange). Error bars indicate the CI95 of the total biomass growth. Different bars within graphs represent FLR subcategories

Natural regeneration was the second most common FLR activity found in the literature (Table 3) and showed the overall third highest potential for carbon sequestration (9.1–18.8 t CO_2_ ha^−1^ year^−1^ for the first 20 years of growth; Table 2). Its removal factors show that biomass growth can be as high or higher in the 20–60-year period after establishment compared to the first 20 years of forest regeneration (Fig. 3a; Table 2). Uncertainty (95% confidence intervals) does not increase as these forest stands age, evidencing a wide age range of data available from naturally regenerated forests exceeding 20-years of age. South America offered the most abundant available data, followed by Central America, yet these data were also the most variable, resulting in a low *R*^2^ (Table 3). Our results show that Africa and Central and South America have similarly high removal factors, while Asia and Oceania, Europe, and North America show similar and lower rates, possibly suggesting a latitudinal (i.e., tropical vs. temperate) driver of biomass growth in this FLR activity. Key gaps in our natural regeneration FLR results are: (i) we did not find published data from African dry forests, and data from the humid forests in Africa were scarce; and (ii) insufficient data were available for Europe to allow differentiating between dry and humid forests.

The evaluation of biomass accumulation for agroforestry FLR yielded removal factors in between those of planted forests and naturally regenerated forests (10.8–15.6 t CO_2_ ha^−1^ year^−1^ for the first 20 years of growth), which could be expected given agroforestry activities typically involve lower planting densities. Removal factors after 20 years, however, are very low (Table 2), with growth rates in the 20–60-year period below 0.1 t C ha^−1^ year^−1^ (Fig. 3b). Overall, agroforestry data were abundant yet the large range of activities that fall under these practices make data highly variable, presenting low *R*^2^ (Table 3). High variability and inconsistent agroforestry types across regions prevented the further subdivision into agroforestry types. While agroforestry FLR is expected to occur across the globe, we did not find sufficient data from Europe, North America, and Oceania, indicating that most publicly available agroforestry studies are performed in developing regions.

Lastly, mangrove restoration FLR, particularly mangrove trees, was found to be highly productive, resulting in the second highest removal factors across all FLR types assessed (23.1 and 10.5 t CO_2_ ha^−1^ year^−1^, the first 20 years of growth and the following 40 years, respectively; Table 2), remaining high throughout the lifetime of mangrove stands (Fig. 3c). Shrub mangroves, on the other hand, had the lowest removal factor of all tropical FLR activities and subcategories and was found to be even lower after 20 years of growth. Our results indicate that mangrove trees are the most commonly planted mangrove type, compared to shrubs (with *n* = 50 and *n* = 13, respectively), and that plantings, overall, are more common in the tropical coasts rather than subtropical ones (Table 3).

## Discussion

### Biomass growth rates across climates and over time

**Fig. 4:**
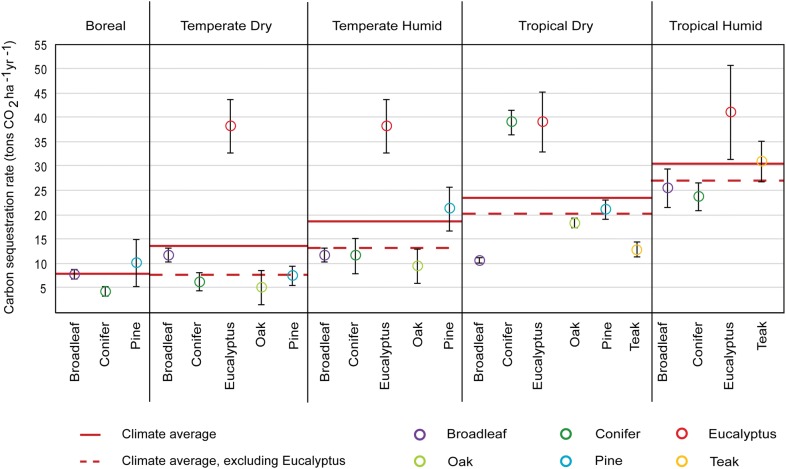
Carbon sequestration rate (tons CO_2_ ha^−1^ year^−1^) of species planted across boreal, temperate, and tropical climates. Each circle represents the average total biomass (above- and belowground) growth rate of the species indicated for a given climate. Error bars indicate the CI95 of their average total biomass growth. The continuous red lines across climates represent the average biomass growth rate for the species included in each climate category, while the dotted red lines represent the average biomass growth rate for all the species in that climate excluding Eucalyptus

Planted forests and natural regeneration were found to be the FLR types that have been most widely studied across the globe, representing 45% and 32% of all the data points collected, respectively, and covering all climates and continents. Both FLR activities demonstrated regional trends, but the variability of natural regeneration across regions, as well as stand age heterogeneity, precluded us from drawing stronger conclusions for this interpretation. We explored, however, the distribution of total biomass growth rates of species planted in planted forests. Our data show that the average growth rate is significantly different between climates (*p*-value < 0.05, *F* (1, 44) = 4.062), consistently increasing from colder to warmer climates, with a stronger relationship when Eucalyptus is excluded (*R*^2^ = 0.99 and *p*-value < 0.001 with Eucalyptus; *R*^2^ = 0.94 and *p*-value < 0.01 without Eucalyptus; Fig. 4). Eucalyptus was found to maintain a relatively consistent growth rate from temperate to tropical climates, dry or humid, evidencing its efficient biomass productivity across regions.

Planted forests, and especially commercial timber plantations, are typically managed to maximize productivity in short periods of time, i.e., during the first 20-year period after establishment [30, 33, 44] and therefore, longer rotation periods would result in lower average sequestration rates (as reflected in CO2FIX carbon modelling program [45]). On the other hand, removal factors produced from our research on natural regeneration show that biomass growth can be as high or higher in the 20–60-year period after establishment (Fig. 3a). Crouzeilles et al. [46] showed similar results in naturally regenerated forests and determined that the diversity among natural forest species and the frequently low initial tree density associated with unassisted natural regeneration favors slow initial biomass accumulation rates of low wood density trees, while succeeding trees establish more consistently and at higher density and with higher wood density once the forest is more established [47]. Agroforestry, in contrast, shows minimum growth after 20 years, suggesting that this FLR category uses fast growing species to maximize the efficiency of this system to provide protection, and/or produce fodder, fruit, or timber, among other products.

### Applicability of this database

#### Accounting for the benefits of forest landscape restoration

The loss and degradation of forest habitat diminishes the ability of the landscape to capture atmospheric CO_2_ and results in the decline of goods and services upon which a significant portion of the global population depend [10, 11], impeding livelihoods and the ability to adapt to a changing climate. Key ecosystem services provided by forest ecosystems include regulation of water quality and quantity [10, 48, 49], regulation of climate [50, 51], protection of biodiversity and soils [12, 13, 52], and provision of food and goods [10, 11, 13]. While forest landscape restoration is not intended as an alternative to conserving forests [53], it can recover multiple benefits lost by deforestation and forest degradation [10, 11, 13, 54] by restoring forest health and ecological functionality at the landscape scale [13, 19, 54].

Despite the multitude of benefits offered by FLR, it is the ability to sequester carbon through the increase of standing biomass in the landscape what has driven many efforts to expand FLR efforts worldwide [19]. Our set of CO_2_ removal factors can be a valuable resource for countries, practitioners and policy makers that need to associate reliable carbon capture numbers with current and planned FLR activities, and thereby help boost global FLR efforts. Where maximizing CO_2_ removal is a priority, the factors provided in this study help identify the most efficient FLR options for capturing carbon in each region, which is shown in this study to be planted forests and woodlots. These monocultures may allow for fast sequestration and potential long-term storage of carbon [10], but can have negative implications for water availability, biodiversity, and other ecosystem functions [15, 55–57] that offer a great range of long-lasting socioenvironmental benefits [13, 54, 58]. Globally, forest species richness has been demonstrated to increase productivity to the extent that the economic value of the forest has been estimated to be over five times the costs of its conservation [28, 29].

Planning FLR actions is approached at the landscape scale, encompassing entire watersheds, diverse land uses, and communities and their livelihoods [13, 19, 54], seeking to modify poor land use practices that led to the loss of forest habitats and landscape fragmentation and to enhance human well-being [12, 13, 19]. Successful FLR approaches must therefore take into consideration the needs and priorities of local communities, balancing the full range of benefits offered by the various FLR options rather than aiming to maximize one [10, 19], and accounting for the viability of FLR activities in the geographic and biophysical context [13, 53]. Accordingly, ensuring successful long-term FLR efforts needs to include parallel efforts to develop a sustainable forest sector and sustainable biomass energy production activities [15]. This entails meeting the long-term needs of populations while introducing campaigns that educate and show communities the value of FLR efforts for the often-overlooked critical ecosystem services they provide. National definitions and circumstances can dictate what activities are officially classified as forestry or agriculture, often influencing which activities are included under national FLR restoration pledges. Where agroforestry is officially considered by governments to be an agricultural practice, it may be excluded from the restoration pledges. However, agroforestry is a valid agriculture-based FLR activity [19] that affords significant socioeconomic and biophysical benefits [54, 58]. The value of this study comes in both providing data for current FLR pledges as well as contributing to the body of knowledge on the mitigation potential of agroforestry activities to support meeting restoration-related goals.

#### The need for robust and specific CO_2_ removal factors

Global commitments such as the Bonn Challenge and NDCs will require reporting on progress made toward established goals. The research undertaken in this study and resulting removals rates offer a useful resource for generating credible estimates of CO_2_ sequestration achieved for countries who made restoration pledges under these commitments and may help inform ongoing efforts by providing a way to compare the relative impact of different FLR activities. Our study also provides pledgers with key data needed to fill current knowledge gaps on removals from FLR activities across regions and climates, and to support their reporting under the Bonn Challenge Barometer of Progress that is currently under development [59]. Further, the CO_2_ removal rates developed in this study may also help analysts to better validate and compare assessed climate impacts as reported by pledgers.

To date, the Forest Land chapter of the IPCC Guidelines [20] has been the most widely used source of tier 1 removal factors where in-country data are scarce or not available. Yet these Guidelines only provide removal factors for natural forests and commonly planted species in forest plantations. This leaves out agroforestry and mangrove restoration FLR activities, common around the globe (Fig. 1), which also play a significant role in removing CO_2_ from the atmosphere (Tables 1, 2) and provide multiple ecosystem service benefits [58, 60]. Furthermore, a significant additional shortcoming of the removal rates offered by the IPCC [20] is that, while offering a data range in some cases, they do not offer estimates of uncertainty nor basic standard statistical information and thus, the accuracy of these commonly used CO_2_ removal rates is unknown.

Langner et al. [21] used pan-tropical biomass maps [23, 24] to estimate alternative tier 1 removal rates of tropical forests and determined that IPCC’s biomass growth rates were up to 35% higher than what their map-based method estimated. Nonetheless, when they compared intact forests only, their results were similar to IPCC default rates. Intact forests, however, do not represent the state of most tropical forests [4, 9, 21]. The removal factors for planted forests and naturally regenerated forests from the IPCC Guidelines [20] and our study are comparable, yet as in previous studies [21], we find them consistently higher than our rates (on average, 41% higher in planted forests and 38% higher in naturally regenerated ones). Although the IPCC does not provide natural regeneration rates for Central America, its biomass growth rates in naturally regenerated forests are higher for Africa and South America compared to the other regions, as in our study.

Overall, the comparison of the suite of FLR CO_2_ removal rates developed in our study with the current IPCC removal rates shows that we include a broader range of FLR activities (agroforestry and mangrove restoration in addition to plantations and natural regeneration, currently represented in the IPCC Guidelines), climates (IPCC plantations defaults are only for tropical climates where as we also include boreal and temperate data), and regions (we provide tier 1 defaults for all regions and climates of the world), along with 95% confidence intervals and goodness of fit (*R*^2^), which the IPCC Guidelines are currently lacking. Our study also represents a more comprehensive and updated compilation of data on tree growth rates, with removals rates derived from data on biomass increment from over 330 published studies and reports, whereas the IPCC 2006 defaults included less than 100 studies (including the 2003 IPCC Guidelines). Further, this study benefits from over 10 years of additional research on biomass carbon sequestration (more than 36% of the studies used in the development of our growth curves were published post-2006).

#### Remaining gaps and limitations

While our set of removal factors (Tables 1, 2) are robust and more comprehensive than those available in the IPCC Guidelines [20], there are still gaps and limitations in data availability. FLR data are available across the globe (Fig. 1), yet a large proportion of FLR studies are focused in tropical regions [15, 33, 61]. As elaborated in the Results section of this paper, these include limited data for some planted species in dry climates, data abundance but high variability of teak and eucalyptus plantations, a lack of data on natural regeneration of African dry forests, scarce data on natural regeneration of European forests, and a limited data available for agroforestry in Europe, North America, and Oceania that has prohibited the construction of specific removal factors for different agroforestry activities. Practitioners seeking to produce CO_2_ removal estimates of agroforestry practices in the United States can also do so using the USDA Comet-Farm [27] platform. CO_2_ removal rates offered by the IPCC Guidelines [20] represent only the aboveground biomass pool, while those produced through our study include both aboveground and a calculated estimate of belowground growth. They therefore exclude other relevant carbon pools in the ecosystem such as soil carbon and, of a lesser size, litter and dead wood carbon pools [62]. While this may underestimate the carbon sequestration potential of FLR activities, the majority of carbon in forest ecosystems is typically stored in living biomass. However, FLR activities such as some restored mangrove forests or forests established in organic soils would have significant soil carbon stocks [63, 64]. Studies seeking to report total ecosystem removal rates would need to include these additional pools, which would require evaluating the availability and applicability of national data, soil carbon maps where available (e.g., soil mangrove carbon map by Sanderman et al. [65]), or using default stocks provided by the IPCC Guidelines [20], with the same limitations mentioned above.

Both IPCC tier 1 defaults and the removal factors developed through our study represent broad, regional estimates that do not necessarily account for important management and biophysical conditions that can significantly impact CO_2_ removals rates. A wide range of factors such as former land use, topography, soil type and quality, microclimate, management practices, and proximity to pollinators and seed sources, can have a significant impact on the success (and subsequent CO_2_ removal) of FLR activities [13, 66–69]. Thus, the removal factors from our study and the IPCC defaults cannot be used as an alternative to site-specific data needed for carbon offset projects seeking to participate in market schemes that transact carbon credits.

Lastly, this study presents the CO_2_ sequestration potential of the assessed FLR activities and does not account for emissions associated with them (e.g., energy inputs). This may be a particularly important shortcoming where associated emissions are significant. For example, silvopastoral agroforestry activities that intensify cattle management can lead to higher methane emissions from enteric fermentation. Where this is the case, it would be necessary to apply additional approaches or tools that account for the net climate impacts of the FLR activities undertaken to ensure complete accounting. In these cases, tools like FAO’s EX-Ante Carbon-balance Tool (EX-ACT) [70], which uses tier 1 defaults, could be used to estimate net emissions or removals.

## Conclusions

Our study represents a comprehensive assessment of CO_2_ removal rates from biomass growth across a wide range of FLR activities that serves as an expansion or update to the widely applied IPCC default tier 1 values. Conducted at the global scale, our review and the removal factors developed offer information on the gross carbon removal potential of four FLR activities across a wide range of climates and species compositions, accompanied by their corresponding values for uncertainty. Further, we provide a complete picture of remaining data gaps and limitations in the existing literature on carbon capture from the atmosphere by FLR activities. The biomass growth curves produced in this study and the removal factors derived from this work may serve as a useful resource for practitioners and decision-makers seeking to better understand the impact of existing or planned FLR actions, especially in data-scarce regions. Alongside the consideration of the needs and priorities of local communities, the unique impact each FLR type has on ecosystem services, and the relative viability of different FLR options in terms of socioeconomic and biophysical conditions, our results provide a valuable input in designing FLR activities to maximize benefits across landscapes and can help boost current FLR efforts worldwide by facilitating reliable carbon capture numbers that can be associated to a wide range of FLR activities.

## Additional files

**Additional file 1.** Figure captions of Additional files 2, and bibliography used to develop the FLR Growth Curves

**Additional file 2.** FLR growth curves

## Abbreviations

FLR: forest landscape restoration
CO_2_: carbon dioxide
GHG: greenhouse gases
IPCC: Intergovernmental Panel on Climate Change
UNFCCC: United Nations Framework Convention on Climate Change
REDD+: reducing emissions from deforestation and forest degradation
NDCs: Nationally Determined Contributions
ROAM: Restoration Opportunities Assessment Methodology
MAI: mean annual increment
CI95: 95% confidence interval

## References

[1] Harris NL, Brown S, Hagen SC, Saatchi SS, Petrova S, Salas W (2012). Baseline map of carbon emissions from deforestation in tropical regions. Science.

[2] Houghton RA (2012). Carbon emissions and the drivers of deforestation and forest degradation in the tropics. Curr Opin Environ Sustain.

[3] Baccini A, Walker W, Carvalho L, Farina M, Sulla-Menashe D, Houghton RA (2017). Tropical forests are a net carbon source based on aboveground measurements of gain and loss. Science.

[4] Pearson TRH, Brown S, Murray LT, Sidman G (2017). Greenhouse gas emissions from tropical forest degradation: an underestimated source. Carbon Balance Manag.

[5] Le Quéré C, Andrew RM, Friedlingstein P, Sitch S, Pongratz J, Manning AC (2018). Global carbon budget 2017. Earth Syst Sci Data.

[6] Hansen MCC, Potapov PV, Moore R, Hancher M, Turubanova SA, Tyukavina A (2013). High-resolution global maps of 21st century forest cover change. Science.

[7] FAO (2016). Global forest resources assessment 2015.

[8] FAO (2018). The state of the world’s forests 2018—forest pathways to sustainable development.

[9] Tyukavina A, Baccini A, Hansen MC, Potapov PV, Stehman SVV, Houghton RA (2015). Aboveground carbon loss in natural and managed tropical forests from 2000 to 2012. Environ Res Lett..

[10] Lamb D, Erskine PD, Parrotta JA (2005). Restoration of degraded tropical forest landscapes. Science.

[11] Sabogal C, Christophe B, McGuire D (2015). Forest and landscape restoration: concepts, approaches, and challenges for implementation. Unasylva.

[12] Aerts R, Honnay O (2011). Forest restoration, biodiversity and ecosystem functioning. BMC Ecol.

[13] Chazdon RL, Uriarte M (2016). Natural regeneration in the context of large-scale forest and landscape restoration in the tropics. Biotropica.

[14] Chazdon RL, Brancalion PHS, Lamb D, Laestadius L, Calmon M, Kumar C (2017). A policy-driven knowledge agenda for global forest and landscape restoration. Conserv Lett.

[15] Chazdon R (2016). Carbon sequestration potential of second-growth forest regeneration in the Latin American tropics. Sci Adv.

[16] UNDP. New York declaration on forests. http://www.undp.org/content/undp/en/home/ourwork/sustainable-development/natural-capital-and-the-environment/biodiversity-and-ecosystems-management/new-york-declaration-on-forests.html. Accessed Sep 2018

[17] Wu A, Gagne C. Aligning ambitions: the case for introducing restoration targets in climate goals. WRI. 2018. https://www.wri.org/blog/2018/09/aligning-ambitions-case-including-restoration-targets-climate-goals. Accessed Oct 2018

[18] IPCC, Masson-Delmotte V, Zhai P, Pörtner HO, Roberts D, Skea J, Shukla PR, et al. Global warming of 1.5 °C. An IPCC special report on the impacts of global warming of 1.5 °C above pre-industrial levels and related global greenhouse gas emission pathways, in the context of strengthening the global response to the threat of climate change. 2018.

[19] IUCN, WRI. A guide to the Restoration Opportunities Assessment Methodology (ROAM) Assessing forest landscape restoration opportunities at the national or sub-national level. The International Union for the Conservation of Nature. http://www.iucn.org/publications. Accessed Sep 2018

[20] IPCC. IPCC guidelines for national greenhouse gas inventories. In: Eggleston S, Buendia L, Miwa K, Ngara T, Tanabe K, editors. Guidelines for national greenhouse gas inventories, vol. 4: agriculture, forestry, and other land use. 2006.

[21] Langner A, Achard F, Grassi G (2014). Can recent pan-tropical biomass maps be used to derive alternative Tier 1 values for reporting REDD+ activities under UNFCCC?. Environ Res Lett.

[22] UNDP, Watson C. Forest carbon accounting: overview and principles. 2009. p. 41.

[23] Saatchi SS, Harris NL, Brown S, Lefsky M, Mitchard ET, Salas W (2011). Benchmark map of forest carbon stocks in tropical regions across three continents. Proc Natl Acad Sci.

[24] Baccini A, Goetz SJ, Walker WS, Laporte NT, Sun M, Sulla-Menashe D (2012). Estimated carbon dioxide emissions from tropical deforestation improved by carbon-density maps. Nat Clim Chang..

[25] Van Deusen P, Heath LS. COLE web applications suite. NCASI and USDA Forest Service, Northern Research Station. http://www.ncasi2.org/COLE/. Accessed Sep 2018

[26] USFS, Dixson GE. The forest vegetation simulator. 2002. https://www.fs.fed.us/fvs/. Accessed Sep 2018

[27] COMET-Farm. http://cometfarm.nrel.colostate.edu/. Accessed Sep 2018

[28] GFBI. Global forest biodiversity initiative database. http://www.gfbinitiative.org/. Accessed Sep 2018

[29] Liang J, Crowther TW, Picard N, Wiser S, Zhou M, Alberti G (2016). Positive biodiversity-productivity relationship predominant in global forests. Science.

[30] Silver WL, Kueppers LM, Lugo AE, Ostertag R, Matzek V (2004). Carbon sequestration and plant community dynamics following reforestation of tropical pasture. Ecol Appl.

[31] Cleveland CC, Townsend AR, Taylor P, Alvarez-Clare S, Bustamante MMC, Chuyong G (2011). Relationships among net primary productivity, nutrients and climate in tropical rain forest: a pan-tropical analysis. Ecol Lett.

[32] Bonner MTL, Schmidt S, Shoo LP (2013). A meta-analytical global comparison of aboveground biomass accumulation between tropical secondary forests and monoculture plantations. For Ecol Manag.

[33] Marin-Spiotta E, Cusack DF, Ostertag R, Silver WL. Trends in aboveground and belowground carbon with forest regrowth after agricultural abandonment in the neotropics. In: Post-agricultural succession in the neotropics. Berlin: Springer; 2008. p. 22–72.

[34] Clark DAJSC (2000). Landscape-scale variation in forest structure and biomass in a tropical rain forest. For Ecol Manag.

[35] Erika M-S, Sharma S (2013). Carbon storage in successional and plantation forest soils: a tropical analysis. Glob Ecol Biogeogr.

[36] Winrock International, IUCN. Global emissions and removals databases—InfoFLR. https://infoflr.org/what-flr/global-emissions-and-removals-databases. Accessed Sep 2018

[37] Zanne AE, Lopez-Gonzalez G, Coomes DA, Ilic J, Jansen S, Lewis SL, Miller RB, Swenson NG, Wiemann MC, Chave J. Global wood density database. Dryad. 2009. https://datadryad.org/pages/repository/. Accessed Sep 2018

[38] Kottek M, Grieser J, Beck C, Rudolf B, Rubel F (2006). World map of the Koppen–Geiger climate classification updated. Meteorol Zeitschrift.

[39] Mitsch WJ, Gosselink JG, Zhang L, Anderson CJ (2009). Wetland ecosystems.

[40] Selvam V, FAO (2007). Tress and shrubs of the Maldives.

[41] Richards FJ (1959). A flexible growth function for empirical use. J Exp Bot.

[42] Pienaar LV, Turnbull KJ (1973). The Chapman-Richards generalization of Von Bertalanffy’s growth model for basal area growth and yield in even-aged stands. For Sci.

[43] Mokany K, Raison RJ, Prokushkin AS (2006). Critical analysis of root:shoot ratios in terrestrial biomes. Glob Chang Biol.

[44] Uri V, Varik M, Aosaar J, Kanal A, Kukumägi M, Lõhmus K (2012). Biomass production and carbon sequestration in a fertile silver birch (*Betula pendula* Roth) forest chronosequence. For Ecol Manag.

[45] Nabuurs GJ, Mohren GMJ (1995). Modelling analysis of potential carbon sequestration in selected forest types. Can J For Res.

[46] Crouzeilles R, Ferreira MS, Chazdon RL, Lindenmayer DB, Sansevero JBB, Monteiro L (2017). Ecological restoration success is higher for natural regeneration than for active restoration in tropical forests. Sci Adv..

[47] Plourde BT, Boukili VK, Chazdon RL (2015). Radial changes in wood specific gravity of tropical trees: inter- and intraspecific variation during secondary succession. Funct Ecol.

[48] Ellison D, Morris CE, Locatelli B, Sheil D, Cohen J, Murdiyarso D (2017). Trees, forests and water: cool insights for a hot world. Glob Environ Chang..

[49] Filoso S, Bezerra MO, Weiss KCB, Palmer MA (2017). Impacts of forest restoration on water yield: a systematic review. PLoS ONE..

[50] Cunningham SC, Mac Nally R, Baker PJ, Cavagnaro TR, Beringer J, Thomson JR (2015). Balancing the environmental benefits of reforestation in agricultural regions. Perspect Plant Ecol Evol Syst.

[51] Dobbs C, Escobedo FJ, Zipperer W (2011). A framework for developing urban forest ecosystem services goods and indicators. Landsc Urban Plan.

[52] Dudley N, Baldock D, Nasi R, Stolton S (2005). Measuring biodiversity and sustainable management in forests and agricultural landscapes. Philos Trans R Soc Lond B Biol Sci.

[53] Holl K, Kappelle M (1999). Tropical forest recovery and restoration. Trends Ecol Evol.

[54] Lamb D, Stanturl J, Madsen P. What is forest landscape restoration? In: Forest landscape restoration: integrating natural and social sciences. Dordrecht: Springer Science + Business Media; 2012. p. 3–23.

[55] Richardson DM, van Wilgen BW, Nunez MA (2008). Alien conifer invasions in South America: short fuse burning?. Biol Invasions.

[56] Brockerhoff EG, Jactel H, Parrotta JA, Quine CP, Sayer J (2008). Plantation forests and biodiversity: oxymoron or opportunity?. Biodivers Conserv.

[57] Bremer LL, Farley KA (2010). Does plantation forestry restore biodiversity or create green deserts? A synthesis of the effects of land-use transitions on plant species richness. Biodivers Conserv.

[58] Perfecto I, Vandermeer J (2008). Biodiversity conservation in tropical agroecosystems: a new conservation paradigm. Ann N Y Acad Sci.

[59] Winrock International, IUCN. Bonn challenge barometer. https://infoflr.org/bonn-challenge/bonn-challenge-barometer/. Accessed Sep 2018

[60] Barbier EB, Hacker SD, Kennedy C, Koch EW, Stier AC, Silliman BR (2011). The value of estuarine and coastal ecosystem services. Ecol Monogr.

[61] Lugo AE (1992). Comparison of tropical tree plantations with secondary forests of similar age. Ecol Monogr.

[62] Liu X, Trogisch S, He J-S, Niklaus PA, Bruelheide H, Tang Z (1885). Tree species richness increases ecosystem carbon storage in subtropical forests. Proc R Soc B Biol Sci.

[63] Donato D, Kauffman JB, Murdiyarso D, Kurnianto S, Stidham M, Kanninen M (2011). Mangroves among the most carbon-rich forests in the tropics. Nat Geosci.

[64] McLeod E, Chmura GL, Bouillon S, Salm R, Björk M, Duarte CM (2011). A blueprint for blue carbon: toward an improved understanding of the role of vegetated coastal habitats in sequestering CO_2_. Front Ecol Environ.

[65] Sanderman J, Hengl T, Fiske G, Solvik K, Adame MF, Benson L (2018). A global map of mangrove forest soil carbon at 30 m spatial resolution. Environ Res Lett.

[66] de Souza FM, Batista JLF (2004). Restoration of seasonal semideciduous forests in Brazil: influence of age and restoration design on forest structure. For Ecol Manag.

[67] Rodrigues RR, Lima RA, Gandolfi S, Nave AG (2009). On the restoration of high diversity forests: 30 years of experience in the Brazilian Atlantic Forest. Biol Conserv.

[68] Campoe OC, Stape JL, Mendes JCT (2010). Can intensive management accelerate the restoration of Brazil’s Atlantic forests?. For Ecol Manag.

[69] Sattler D, Murray LT, Kirchner A, Lindner A (2014). Influence of soil and topography on aboveground biomass accumulation and carbon stocks of afforested pastures in South East Brazil. Ecol Eng.

[70] FAO. EX-ante carbon balance tool (EX-ACT). http://www.fao.org/tc/exact/ex-act-home/en/. Accessed Sep 2018

