# Supplementary material for: Global carbon dioxide removal rates from forest landscape restoration activities
Source: Carbon Balance Manag. 2018 Nov 20;13:22. doi: 10.1186/s13021-018-0110-8 (PMC6246754; doi:10.1186/s13021-018-0110-8)
Supplement: Supplementary file 1 — Additional file 1. Figure captions of Additional files 2, and bibliography used to develop the FLR Growth Curves [file 13021_2018_110_MOESM1_ESM.docx]

# **Additional Materials - Global Carbon Dioxide Removal Rates from Forest Landscape Restoration Activities.**

# **1. FLR Growth Curves**

## 1.1. Planted forests and Woodlots

### 1.1.A. Oak

**Figure 1.1.A.** Growth curves developed for oak (*Quercus* sp.) planted forests and woodlots under temperate humid, temperate dry, and tropical dry climates. The curves indicate biomass carbon stock (tons C ha^-1^) per stand age (years), upper and lower bounds of the 95% confidence interval (CI), and goodness of fit (R^2^).

### 1.1.B. Teak

**Figure 1.1.B.** Growth curves developed for teak (*Tectona grandis*) planted forests and woodlots under tropical humid and tropical dry climates. The curves indicate biomass carbon stock (tons C ha^-1^) per stand age (years), upper and lower bounds of the 95% confidence interval (CI), and goodness of fit (R^2^).

### 1.1.C. Eucalyptus

**Figure 1.1.C.** Growth curves developed for eucalyptus (*Eucalyptus* sp.) planted forests and woodlots under temperate humid and dry, tropical humid, and tropical dry climates. The curves indicate biomass carbon stock (tons C ha^-1^) per stand age (years), upper and lower bounds of the 95% confidence interval (CI), and goodness of fit (R^2^).

### 1.1.D. Broad leaf, excluding eucalyptus and teak

**Figure 1.1.D.** Growth curves developed for planted forests and woodlots of broadleaf excluding teak and eucalyptus (e.g., *Populus* sp., *Gmelina* sp., or *Leucaena* sp., among others), under boreal, temperate humid and dry, tropical humid, and tropical dry climates. The curves indicate biomass carbon stock (tons C ha^-1^) per stand age (years), upper and lower bounds of the 95% confidence interval (CI), and goodness of fit (R^2^).

### 1.1.E. Pine

**Figure 1.1.E.** Growth curves developed for pine (*Pinus* sp.) planted forests and woodlots under temperate humid and dry, tropical humid, and tropical dry climates. The curves indicate biomass carbon stock (tons C ha^-1^) per stand age (years), upper and lower bounds of the 95% confidence interval (CI), and goodness of fit (R^2^).

### 1.1.F. Conifer, excluding pine

**Figure 1.1.F.** Growth curves developed for planted forests and woodlots of conifer excluding pine (e.g., *Cupresssus* sp., *Abies* sp., among others), under boreal, temperate humid and dry, tropical humid, and tropical dry climates. The curves indicate biomass carbon stock (tons C ha^-1^) per stand age (years), upper and lower bounds of the 95% confidence interval (CI), and goodness of fit (R^2^).

## 1.2. Natural Regeneration

### 1.2.A. Asia and Oceania

**Figure 1.2.A.** Growth curves developed for naturally regenerated forests in Asia and Oceania under humid and dry climates. The curves indicate biomass carbon stock (tons C ha^-1^) per stand age (years), upper and lower bounds of the 95% confidence interval (CI), and goodness of fit (R^2^).

### 1.2.B. Europe

**Figure 1.2.B.** Growth curves developed for naturally regenerated forests in Europe under humid and dry climates. The curve indicates biomass carbon stock (tons C ha^-1^) per stand age (years), upper and lower bounds of the 95% confidence interval (CI), and goodness of fit (R^2^).

### 1.2.C. Africa

**Figure 1.2.C.** Growth curves developed for naturally regenerated forests in Africa under humid climate. The curve indicates biomass carbon stock (tons C ha^-1^) per stand age (years), upper and lower bounds of the 95% confidence interval (CI), and goodness of fit (R^2^).

### 1.2.D. North America

**Figure 1.2.D.** Growth curves developed for naturally regenerated forests in North America under humid and dry climates. The curves indicate biomass carbon stock (tons C ha^-1^) per stand age (years), upper and lower bounds of the 95% confidence interval (CI), and goodness of fit (R^2^).

### 1.2.E. Central America and the Caribbean

**Figure 1.2.E.** Growth curves developed for naturally regenerated forests in Central America and the Caribbean under humid and dry climates. The curves indicate biomass carbon stock (tons C ha^-1^) per stand age (years), upper and lower bounds of the 95% confidence interval (CI), and goodness of fit (R^2^).

### 1.2.F. South America

**Figure 1.2.F.** Growth curves developed for naturally regenerated forests in South America under humid and dry climates. The curves indicate biomass carbon stock (tons C ha^-1^) per stand age (years), upper and lower bounds of the 95% confidence interval (CI), and goodness of fit (R^2^).

## 1.3. Agroforestry

### 1.3.A. Africa

**Figure 1.3.A.** Growth curves developed for agroforestry in Africa. The curve indicates biomass carbon stock (tons C ha^-1^) per stand age (years), upper and lower bounds of the 95% confidence interval (CI), and goodness of fit (R^2^).

### 1.3.B. Asia

**Figure 1.3.B.** Growth curves developed for agroforestry in Asia. The curve indicates biomass carbon stock (tons C ha^-1^) per stand age (years), upper and lower bounds of the 95% confidence interval (CI), and goodness of fit (R^2^).

### 1.3.C. Latin America and the Caribbean

**Figure 1.3.C.** Growth curves developed for agroforestry in Latin America and the Caribbean. The curve indicates biomass carbon stock (tons C ha^-1^) per stand age (years), upper and lower bounds of the 95% confidence interval (CI), and goodness of fit (R^2^).

## 1.4. Mangrove Restoration

### 1.4.A. Mangrove trees

**Figure 1.4.A.** Growth curves developed for restoration of mangrove trees. The curve indicates biomass carbon stock (tons C ha^-1^) per stand age (years), upper and lower bounds of the 95% confidence interval (CI), and goodness of fit (R^2^).

### 1.4.B. Mangrove shrubs

**Figure 1.4.B.** Growth curves developed for restoration of mangrove shrubs. The curve indicates biomass carbon stock (tons C ha^-1^) per stand age (years), upper and lower bounds of the 95% confidence interval (CI), and goodness of fit (R^2^).

# **2. Bibliography of FLR Growth Curves**

## 2.1. Planted forests and Woodlots

Borja, I., H. de Wit, A. Steffenrem, H. Majdi. 1998. Stand Age and Fine Root Biomass, Distribution and Morphology in a Norway Spruce Chronosequence in Southeast Norway. Tree Physiology 28: 773-84.

Brown, A. G., E. K. S. Nambier, C. Cossalter. 1997. Plantations for the tropics: their role extent and nature. In: Nambiar, E. K. S. and Brown, A. (eds). Management of soil, water and nutrients in Tropical Plantation Forest, p 1-23. Australian Centre for International Agricultural research (ACIAR). Monograph 43. Canberra.

Burcary, B. J. R. 1967. El Ciprés (*Cupressus lusitanica* Mill.) como base de las reforestaciones planificadas en el Valle Central de Costa Rica. Tesis Mag. Sc. Turrialba, Costa Rica. IICA. 102 pp.

Cairns, M. A., S. Brown, E. H. Helmer, G. A. Baumgardner. 1997. Root Biomass Allocation in the World’s Upland Forests. Oecologia 111: 1-11.

Camargo, J. C., J. A. Rodríguez, A. M. Arango. 2010. Crecimiento y fijación de carbono en una plantación de guadua en la zona cafetera de Colombia. Recursos Naturales y Ambiente 61: 86-94.

Chavarria, E. M. I. 1996. Curvas preliminares de índices de sitio y factores asociados con el crecimiento del *Eucalyptus deglupta* Blume (deglupta) para la Región Huetar Norte en Costa Rica. Tesis Licenciatura. Universidad Nacional (UNA). C. R. 100 pp.

Chen, Y., A. Z. Liu, X. Rao, X. Wang, C. Liang, Y. Lin, L. Zhou, X. Cai, S. Fu. 2015. Carbon Storage and Allocation Pattern in Plant Biomass among Different Plantation Stands in Guyandong, China. Forests 6: 794-808.

Christie, S. I., R. J. Scholes. 1995. Carbon Storage in Eucalyptus and Pine Plantations in South Africa. Environmental Monitoring and Assessment 38: 231–241.

Du Toit, B. 2008. Effects of Site Management on Growth, Biomass Partitioning and Light Use Efficiency in a Young Stand of Eucalyptus Grandis in South Africa. Forest Ecology and Management 255: 2324–2336.

Dye, P. J., S. Jacobs, D. Drew. 2004. Verification of 3-PG Growth and Water-Use Predictions in Twelve Eucalyptus Plantation Stands in Zululand, South Africa. Forest Ecology and Management 193: 197–218.

Evans, J. 1992. Plantation forestry in the tropics. Second edition. Oxford Science Publication. USA. 403 p.

FOOD AND AGRICULTURE ORGANIZATION (FAO). 2001. Mean annual volume increment of selected industrial forest plantation species. L. Ugalde, O. Pérez (eds.). Forest Plantation Thematic Papers, Working Paper 1. Forest Resources Development Service, Forest Resources Division. FAO, Rome.

Fleming, R. L., A. D. Smith. 2011. Examining 40-year stand development following vegetation control: white spruce planted in a trembling aspen dominated cutover. Can. J. For. Res. 41: 728–739.

Fonseca, W., F. E. Alice, J. M. Rey-Benayas. 2012. Carbon Accumulation in Aboveground and Belowground Biomass and Soil of Different Age Native Forest Plantations in the Humid Tropical Lowlands of Costa Rica. New Forests 43: 197-211.

Gurumurti, K., D. P. Raturi, H. C. S. Bhandari. 1984. Biomass production in energy plantations of *Prosopis juliflora*. Indian For. 110: 879-894.

Hillis, W. E., Brown A. G. 1984. Eucalypts for wood production. CSIRO/Academic Press. London. 434 pp.

Hunt, S. L., A. M. Gordon, D. M. Morris. 2010. Carbon Stocks in Managed Conifer Forests in Northern Ontario, Canada. Silva Fennica 44: 563-582.

Hynynen, J., P. Niemistö, A. Viherä-Aarnio, A. Brunner, S. Hein, and P. Velling. 2010. Silviculture of Birch (*Betula Pendula* Roth and *Betula Pubescens* Ehrh.) in Northern Europe. Forestry 83: 103–119.

INFOR. 1986. Especies forestales exóticas de interés económico para Chile. Santiago, Chile. Gerencia de Desarrollo, CORFO AF 86/32. 167 pp.

Jha, K. K. 2015. Carbon Storage and Sequestration Rate Assessment and Allometric Model Development in Young Teak Plantations of Tropical Moist Deciduous Forest, India. Journal of Forestry Research 26: 589-604.

Justine, M. F., W. Yang, F. Wu, B. Tan, M. N. Khan, Y. Zhao. 2015. Biomass Stock and Carbon Sequestration in a Chronosequence of *Pinus Massoniana* Plantations in the Upper Reaches of the Yangtze River. Forests 6: 3665-3682.

Kaul, O. N., D. C. Sharma, V. N. Tandon. 1983. Biomass distribution and productivity in a poplar plantation. Indian For. 109: 822-828.

Kraenzel, M., A. Castillo, T. Moore, C. Potvin. 2003. Carbon Storage of Harvest-Age Teak (*Tectona Grandis*) Plantations, Panama. Forest Ecology and Management 173: 213–25.

Ladrach, W. E. 1986. Thinning of *Pinus patula* by mechanical and selective method: results at 10 years. In: Whitmore, J. L., N. F. de Barros, R. Salazar (eds). Plantation Forests for Wood Production in the Neotropics, pp. 17. Abstracts of Three IUFRO/MAB Symposia. Costa Rica.

Lamprecht, H. 1990. Silviculture in the Tropics: tropical forest ecosystems and their tree species-possibilities and methods for their long-term utilization. GTZ, Eschborn. Germany. 296 pp.

Lasco, R. D. 2002. Forest carbon budgets in Southeast Asia following harvesting and land cover change. Science in China 45: 55–64.

Lodhiyal, N., L. S. Lodhiyal, Y. P. S. Pangtey. 2002. Structure and Function of Shisham Forests in Central Himalaya, India: Dry Matter Dynamics. Ann Bot. 89: 41-54.

Long, J. N., J. Turner. 1975. Aboveground Biomass of Understory and Overstory in an Age Sequence of Four Doughlas-Fir Stands. Journal of Applied Ecology 12: 179.

Lugo, A. E., D. Wang, F. H. Bormann. 1990. A Comparative Analysis of Biomass Production in Five Tropical Tree Species. Forest Ecology and Management 31: 153-66.

Lugo, A. E. 1992. Comparison of Tropical Tree Plantations with Secondary Forests of Similar Age. Ecological Monographs 62: 1-41.

Madgwick, H. A. I., D. S. Jackson, P. J. Knight. 1977. Above-ground dry matter, energy, and nutrient contents of trees in an age series of *Pinus radiata* plantations. New Zealand Journal of Forest Science 7: 445-468.

Marin-Spiotta, E., D. F. Cusack, R. Ostertag, W. L. Silver. 2008. Trends in Aboveground and Belowground Carbon with Forest Regrowth After Agricultural Abandonment in the Neotropics. In: Post-Agricultural Succession in the Neotropics, pp. 22-72.

NATIONAL ACADEMY OF SCIENCES (NAS). 1983. Firewood crops: shrub and tree species for energy production. National Academy Press, Washington, D. C. Vol. 2. 92 pp.

Navarro, C. 1985. Producción de biomasa de *Eucalyptus deglupta* en una plantación de ocho años en Turrialba, Costa Rica. Silvoenergia 8: 4 pp.

Negi, J. D. S., N. K. S. Bora, V. N. Tandon, H. D. Thapliyal. 1984. Organic matter production in an age series of *Eucalyptus globulus* plantations in Tamil Nadu. Indian For. 110: 802-813.

Nerkar, V. G., 1984. Irrigated subabul plantations in Yavatmal District for raising biomass. Indian For. 110: 861-867.

Newman, D. 1981. Third year growth of the species in the Pulpapel Arboretum. Research Report, Investigación Forestal, Cartón de Colombia, No. 66. 7 pp.

Nunes, L., D. Lopes, F. Castro Rego, S. T. Gower. 2013. Aboveground Biomass and Net Primary Production of Pine, Oak and Mixed Pine-Oak Forests on the Vila Real District, Portugal. Forest Ecology and Management 305: 38-47.

Ola-Adams, B. A., 1976. Dry matter production and nutrient content of a stand of coppiced *Cassia siamea* Lam. in Ibadan fuel plantation. Nigeria J. For. 6: 63-66.

Oliveira, N., H. Sixto, I. Cañellas, R. Rodríguez-Soalleiro, C. Pérez-Cruzado. 2015. Productivity Model and Reference Diagram for Short Rotation Biomass Crops of Poplar Grown in Mediterranean Environments. Biomass and Bioenergy 72: 309-320.

Oren, R., E. D. Schulze, K. S. Werk, J. Meyer, B. U. Schneider, H. Heilmeier. 1988. Performance of two *Picea Abies* (L.) karst stands at different stages of decline - I. Carbon relations and stand growth. Oecologia 75: 25-37.

Otarola, A., L. Ugalde. 1989. Tablas de volumen para *Eucalyptus camaldulensis* en Nicaragua. Proyecto IRENA-CATIE-ROCAP No. 596-0089. Turrialba, C. R. 14 pp.

Parrotta, J. A., O. H. Knowles. 1999. Restoration of Tropical Moist Forests on Bauxite-Mined Lands in the Brazilian Amazon. Restoration Ecology 7: 103–116.

Peichl, M., M. A. Arain. 2007. Allometry and partitioning of above- and belowground tree biomass in an age-sequence of white pine forests. Forest Ecology and Management 6: 72-81.

Peichl, M., A. M. Arain, T. R. Moore, J. J. Brodeur, M. Khomik, S. Ullah, N. Restrepo-Coup, J. McLaren, M. R. Pejam. 2014. Carbon and greenhouse gas balances in an age sequence of temperate pine plantations. Biogeosciences 11: 5399-5410.

Pérez-Cruzado, C., A. Merino, R. Rodríguez-Soalleiro. 2011. A management tool for estimating bioenergy production and carbon sequestration in *Eucalyptus Globulus* and *Eucalyptus Nitens* grown as short rotation woody crops in North-West Spain. Biomass and Bioenergy35: 2839-2851.

Prasad, R., A. K. Sah, A. S. Bhandari, O.P. Choubey. 1984. Dry matter production by *Eucalyptus camaldulensis* Dehn plantation in Jabalpur. Indian For. 110: 868-878.

Raman, S. S. 1976. Biological productivity of Shorea plantations. Indian For. 102: 174-184.

Ray, M. P. 1971. Plantations of *Casuarina equisetifolia* in the Midnapore district, West Bengal. Indian Forester 97: 443-457.

Redondo-Brenes, A., F. Montagnini. 2006. Growth, productivity, aboveground biomass, and carbon sequestration of pure and mixed native tree plantations in the Caribbean lowlands of Costa Rica. Forest Ecology and Management 232: 168-178.

Rodrigues Pereira, A., D. Coutinho de Andrade, P. G. Lelis Leal, N. C. dos Santos Teixeira. 1984. Producao de biomassa e remocao de nutrientes em povoamentos de *Eucalyptus citriodora* e *Eucalyptus saligna* cultivados na regiao de Cerrado de Minas Gerais. Rev. Flor. 15: 8-16.

Rose, D., R. Salazar. 1983. Cuantificación de la producción de Lefia en un rodal de *Gmelina arborea* Roxb. y Hojancha. CATIE, Turrialba, Costa Rica.

Ruark, G. A., J. G. Bockheim. 1987. Biomass, net primary production, and nutrient distribution for an age sequence of *Populus Tremuloides* ecosystems. Canadian Journal of Forest Research 18: 435-443.

Rytter, L., R. M. Rytter. 2016. Growth and carbon capture of grey alder (*Alnus Incana* (L.) Moench.) under North European conditions - Estimates based on reported research. Forest Ecology and Management 399: 82-93.

Salazar, R., 1985. Productividad del *Pinus caribaea* var. hondurensis Barr. y Golf. en Turrialba, Costa Rica. IPEF, Paracicaba 29: 19-24.

Sharrow, S. H., S. Ismail. 2004. Carbon and nitrogen storage in agroforests, tree plantations, and pastures in western Oregon, USA. Agroforestry Systems 60: 123-130.

Silver, W. L., L. M. Kueppers, A. E. Lugo, R. Ostertag, V. Matzek. 2004. Carbon sequestration and plant community dynamics following reforestation of tropical pasture. Ecological Applications 14: 1115-27.

Singh, R. P. V. K. Sharma. 1976. Biomass estimation in five different aged plantations of *Eucalyptus tereticornis* Smith in Western Uttar Pradesh. In: XVI International Congress of IUFRO, Oslo, Norway, 22 June 1976. Univ. Maine Press, Orono, pp. 143-161.

Singh, R. P. 1982. Net primary productivity and productive structure of *Eucalyptus tereticornis* Smith plantations grown in Gangetic Plain. Indian For., 108: 261-269.

Smith, J. E., L. S. Heath, K. E. Skog, R. A. Birdsey. 2006. Methods for calculating forest ecosystem and harvested carbon with standard estimates for forest types of the United States. USDA Forest Service General Technical Report NE-343. 222 pp.

Sommer, A., T. Dow. 1978. Compilation of indicative growth and yield data of fast growing exotic tree species planted in tropical and subtropical regions. FAO. 75 pp.

Son, Y., S. T. Gower. 1991. Aboveground Nitrogen and Phosphorus Use by Five Plantation-Grown Trees with Different Leaf Longevities. Biogeochemistry 14: 167-191.

Sulistyawati E., Y. I. Ulumudding, D. M. Hakim, A. B. Harto, M. Ramdhan. 2006. Estimation of carbon stock at landscape level using remote sensing: a case study in Mount Papandayan. Proceedings of the Environmental Technology and Management Conference, Bandung, West Java.

Turnbull, J. W. 1983. The use of *Casuarina equisetifolia* for protection forest in China. In: Midgley, S. J., J. W. Turnbull, R. D. Johnston (eds.), Casuarina Ecology, Management and Utilization. CSIRO Division of Forest Research, Canberra, pp. 55-57.

Uri, V., M. Varik, J. Aosaar, A. Kanal, M. Kukumgi, and K. Lhmus. 2012. Biomass production and carbon sequestration in a fertile silver birch (*Betula Pendula* Roth) forest chronosequence. Forest Ecology and Management 267: 117-126.

Vanninen, P., H. Ylitalo, R. Sievanen, A. Makela. 1996. Effects of age and site quality on the distribution of biomass in scots pine (*Pinus Sylvestris* L.). Trees 10: 231-38.

Vasquez, W., L. Ugalde. 1994. Rendimiento y calidad de sitio para *Gmelina arborea, Tectona grandis, Bombacopsis quinata* y *Pinus caribaea* en Guanacaste, Costa Rica. Informe Final Convenio de Cooperación, Proyecto Forestal Chorotega (IDA/FAO) y Proyecto Madelaña-3/CATIE. 42 pp.

Wadsworth, F. H. 1997. Forest Production for Tropical America. USDA. Forest Service. Agriculture Handbook 710. USA. 563 pp.

Wang, F., X. Xu, B. Zou, Z. Guo, Z. Li, W. Zhu. 2013. Biomass accumulation and carbon sequestration in four different aged *Casuarina Equisetifolia* coastal shelterbelt plantations in South China. PLoS ONE 8: e77449.

Zhang, H., T. Song, K. Wang, H. Du, Y. Yue, G. Wang, F. Zeng. 2014. Biomass and carbon storage in an age-sequence of *Cyclobalanopsis glauca* plantations in southwest China. Ecological Engineering 73: 184-191.

Zhao, M., W. Xiang, C. Peng, D. Tian. 2009. Simulating age-related changes in carbon storage and allocation in a Chinese fir plantation growing in Southern China using the 3-PG Model. Forest Ecology and Management 257: 1520-1531.

## 2.2. Natural Regeneration

Aide, T. M., J. Z. Zimmerman, H. Herrera, M. Rosario, M. Serrano. 1995. Forest recovery in abandoned tropical pastures in Puerto Rico. Forest. Ecol. Manag. 77: 77-86.

Alves, L. F., S. A. Vieira, M. A. Scaranello, P. B. Camargo, F. A. M. Santos, C. A. Joly, L. A. Martinelli. 2010. Forest structure and live aboveground biomass variation along an elevational gradient of tropical Atlantic moist forest (Brazil). Forest Ecology and Management 260: 679-91.

Alves, D. S., J. V. Soares, S. Amaral, E. M. K. Mello, S. A. S. Almeida, O. Fernandes da Silva, A. M. Silveira. 1997. Biomass of primary and secondary vegetation in Rondonia, Western Brazilian Amazon. Global Change Biology 3: 451-461.

Bartholomew, W. V., J. Meyer, H. Laudelot. 1953. Mineral nutrient immobilization under forest and grass fallow in the Yangambi (Belgian Congo) region. Publications de L'Institut National pour L'Étude Agronomique du Congo Beige, Série Scientifique No. 57, 27 pp.

Cañellas, I., M. Sánchez González, S. M. Bogino, P. Adame, C. Herrero, S. Roig, M. Tomé, J. A. Paulo, F. Bravo. 2008. Silviculture and carbon sequestration in Mediterranean oak forests. Managing Forest Ecosystems: The Challenge Climate Change 17: 317338.

Chen, Y., Z. Liu, X. Rao, X. Wang, C. Liang, Y. Lin, L. Zhou, X. Cai, S. Fu. 2015. Carbon storage and allocation pattern in plant biomass among different forest plantation stands in Guangdong, China. Forests 6: 794-808.

Davidson, E. A., C. J. R. de Carvalho, I. C. G. Vierira, R. O. Figuereido, P. Moutinho, F. Y. Ishida, M. T. P. dos Santos, J. B. Guerrero, K. Kalif, R. T. Saba. 2004. Nitrogen and phosphorous limitation of biomass growth in a tropical secondary forest. Ecological Applications 14: S150-S163.

de Koning, G. H. J., E. Veldkamp, M. Lopez-Ulloa. 2003. Quantification of carbon sequestration in soils following pasture to forest conversion in northwestern Ecuador. Global Biogeochemical Cycles 17: 1098.

del Valle, J. I., H. I. Restrepo, M. M. Londono. 2011. Recuperación de la biomasa mediante la sucesión secundaria, Cordillera Central de los Andes, Colombia. Rev. Biol. Trop. 59: 1337-1358.

Drew, W. B., S. Aksornkoae, W. Kaitpraneet. 1978. An assessment of productivity in successional stages from abandoned swidden (rai) to dry evergreen forest in northeastern Thailand. Forest Research Bulletin 56, Kasetsart University, Bangkok.

Eaton J.M. and D. Lawrence. 2006. Woody debris stocks and fluxes during succession. Forest Ecology and Management 232: 46-55.

Ewel, J. J. 1971. Biomass changes in early tropical succession. Turrialba 21: 110–112.

Ewel, J. J. 1976. Litterfall and leaf decomposition in a tropical forest succession in eastern Guatemala. The Journal of Ecology 64: 293-308.

Ewel, J. J. 1983. Succession. In: Tropical rainforest ecosystems. Golley, F. B. (eds). Elsevier Scientific Publishing Co., Amsterdam, The Netherlands. Pp. 217–223.

Faber-Langendoen, D., A. H. Gentry. 1991. The structure and diversity of rain forests at Bajo Calima, Chocó Region, Western Colombia. Biotropica 23: 2-11.

Folster, H., G. de las Salas, P. Khanna. 1976. A tropical evergreen forest site with perched water table, Magdalena Valley, Colombia: biomass and bioelement inventory of primary and secondary vegetation. Oecologia Planetarium 11: 297-320.

Fonseca W., F. E. Alice, J. M. Rey-Benayas. 2012. Carbon accumulation in aboveground and belowground biomass and soil of different age native forest plantations in the humid tropical lowlands of Costa Rica. New Forests 43: 197–211.

Gehring, C., M. Denich, M. Kanashimo, P.L.G. Vlek. 1999. Response of secondary vegetation in Eastern Amazonia to relaxed nutrient availability constraints. Biogeochemistry 45: 223-241.

Gehring C., M. Denich, P. L. G. Vlek. 2005. Resiliency of secondary forest regrowth after slash-and-burn agriculture in central Amazonia. Journal of Tropical Ecology 21: 519-527.

Greenland, D. J., and J. M. L. Kowal. 1960. Nutrient content of the moist tropical forest of Ghana. Plant and Soil 12: 154–174.

Gries, J. F. 1995. Biomass and net primary production for a northern hardwood stand developmental sequence in the Upper Peninsula, Michigan. Thesis, University of Wisconsin-Madison, Madison, WI, USA.

Hughes, R. F., J. B. Kauffman, V. J. Jaramillo. 1999. Biomass, carbon, and nutrient dynamics of secondary forests in a humid tropical region of Mexico. Ecology 80: 1892-1907.

Jepsen, M. R. 2006. Above-ground carbon stocks in tropical fallows, Sarawak, Malaysia. Forest Ecology and Management 225: 287-295.

Johnson, C. M., I. C. G. Vieira, D. J. Zarin, J. Frinzano, A. H. Johnson. 2001. Carbon and nutrient storage in primary and secondary forests in eastern Amazonia. Forest Ecology and Management 147: 245-250.

Jordan, C. F., E. G. Farnsworth. 1982. Natural vs plantation forests: a case study of land reclamation strategies for the humid tropics. Environmental Management 6: 485-492.

Kenzo, T., T. Ichie, D. Hattori, J. J. Kendawang, K. Sakurai, I. Ninomiya. 2010. Changes in above- and belowground biomass in early successional tropical secondary forests after shifting cultivation in Sarawak, Malaysia. Forest Ecology and Management 260: 875–882.

Léonardi, S., M. Rapp, A. Dénes. 1992. Biomasse, minéralomasse, productivité et gestion de certains éléments biogènes dans une forêt de *Quercus suber* L. en Sicile (Italie). Ecologia Méditerranea 18: 89–98.

Litton, C. M., M. G. Ryan, D. B. Tinker, D. H. Knight. 2003. Belowground and aboveground biomass in young postfire lodgepole pine forests of contrasting tree density. Can. J. For. Res. 33: 351–363.

Long, J. N., J. Turner. 1975. Aboveground biomass of understory and overstory in an age sequence of four douglas-fir stands. Ecology 53: 235-242.

Lucas, R. M., M. Honzak, I. do Amaral, P. J. Curran, G. M. Foody. 2002. Forest regeneration on abandoned clearances in central Amazonia. International Journal of Remote Sensing 23: 965-988.

Lucas, R. W, R. Salguero-Gomez, D. B. Cobb, B. G. Waring, F. Anderson, W. J. McShea. 2013. White-tailed deer (*Odocoileus virginianus*) positively affect the growth of mature northern red oak (*Quercus rubra*) trees. Ecosphere 4: 84 (17 p).

Lugo, A. E. 1992. Comparison of tropical tree plantations with secondary forests of similar age. Ecological Monographs 62: 1-41.

Marin-Spiotta E., R. Ostertag, W. L. Silver. 2007. Long-term patterns in tropical reforestation: plant community composition and aboveground biomass accumulation. Ecological Applications 17: 828-839.

Marin-Spiotta, E., D. F. Cusack, R. Ostertag, W. L. Silver. 2008. Trends in above and belowground carbon with forest regrowth after agricultural abandonment in the neotropics. In: Post-Agricultural Succession in the Neotropics, R. W. Myster (eds). Springer, p. 22-72.

Maury-Lechon, G. 1982. Regeneration forestière en Guyana française: recru sur 25 ha de coupe papetière en forêt dense humide (Arbocel.). Revue Boiset des Tropiqus 197: 3-21.

Norton, S. A., H. E. Young. 1976. Forest biomass utilization and nutrient budgets. In: Oslo Biomass Studies. H. E. Young (eds). College of Life Sciences and Agriculture, University of Maine, Orono, ME, p. 55-73.

Nye, P. H., D. J. Greenland. 1960. The soil under shifting cultivation. Technical Comment No. 51, Commonwealth Bureau of Soils, Harpenden, England.

Omeja, P. A., C. A. Chapman, J. Obua. 2009. Enrichment planting does not improve tree restoration when compared to natural regeneration in a former pine plantation in Kibale National Park, Uganda. Afr. J. Ecol. 47: 650–657.

Pajtik, J., B. Konopka, M. Lukac. 2008. Biomass functions and expansion factors in young Norway spruce (*Picea abies* L. Karst) trees. Forest Ecology and Management 256: 1096–1103.

Read, L., D. Lawrence. 2003. Recovery of biomass following shifting cultivation in dry tropical forests of the Yucatan. Ecological Applications 13: 85-97.

Ruark, G.A., J.G. Bockheim. 1988. Biomass, net primary production, and nutrient distribution for an age sequence of *Populus tremuloides* ecosystems. Can. J. For. Res. 18: 435-443.

Sabhasri, S. 1978. Effects of forest fallow cultivation on forest production and soil. In: Economic development and marginal agriculture in northern Thailand. Kunstadter, P., E. C. Chapman, S. Sabhasri (eds). University Press of Hawaii, Honolulu, p. 160–184.

Saldarriaga, J. G., D. C. West, M. L. Thorp. 1986. Forest succession in the Upper Rio Negro of Colombia and Venezuela. Environmental Sciences Division Publication No. 2694 (ORNL/TM-9712), Oak Ridge National Laboratory, Oak Ridge, TN.

Saldarriaga, J. G., D. C. West, M. L. Tharp, C. Uhl. 1988. Long-term chronosequence of forest succession in the upper Rio Negro of Colombia and Venezuela. Journal of Ecology 76: 938-958.

Salimon, C. I., I. F. Brown. 2000. Secondary forests in wester Amazonia: significant sinks for carbon released from deforestation. Interciencia 25: 198-202.

Scheller, R. M., D. J. Mladenoff. 2004. A forest growth and biomass module for a landscape simulation model, LANDIS: design, validation, and application. Ecological Modelling 180: 211-229.

Schroth, G., S. A. D’Angelo, W. G. Teixeira, D. Haag, R. Lieberei. 2002. Conversion of secondary forest into agroforestry and monoculture plantations in Amazonia: consequences for biomass, litter and soil carbon stocks after 7 years. Forest Ecology and Management 163: 131-150.

Sebei, H., A. Albouchi, M. Rapp, M. H. El Aouni. 2001. Évaluation de la biomasse arborée et arbustive dans une séquence de degradation de la suberaie à Cytise de Kroumirie (Tunisie). Annals. Forest Sci. 58: 175–191.

Silver, W. L., F. N. Scatena, A. H. Johnson, T. G. Siccama, F. Watt. 1996. At what temporal scales does disturbance affect belowground nutrient pools? Biotropica 28: 441-457.

Singh, R. P. 1975. Biomass, nutrient and productivity structure of a stand of dry deciduous forest of Varanasi. Tropical Ecology 16: 104–109.

Smith Jr, C. T., M. L. McCormack Jr, J. W. Hornbeck, C. W. Martin. 1986. Nutrient and biomass removals from a red spruce-balsam fir whole tree harvest. Can. J. For. Res. 16: 381-388.

Spracklen, D. V., R. Righelato. 2016. Carbon storage and sequestration of re-growing montane forests in southern Ecuador. Forest Ecology and Management 364: 139-144.

Szott, T., C. A. Palm, C. B. Davey. 1994. Biomass and litter accumulation under managed and natural tropical fallows. Forest Ecology and Management 67: 177-190.

Tergas, L. E., H. C. Popenoe. 1971. Young secondary vegetation and soil interactions in Izabal, Guatemala. Plant and Soil 34: 675-690.

Toky, O. P., P. S. Ramakrishnan. 1983. Secondary Succession Following Slash and Burn Agriculture in North- Eastern India: II. Nutrient Cycling. Journal of Ecology 71: 747-757.

Tran, T. N., G. P. Shivakoti, M. Inoue. 2010. Changes in property rights, forest use and forest dependency of Katu communities in Nam Dong District, Thua Thien Hue Province, Vietnam. Int. For. Rev. 12: 307–319.

Uhl, C., C. Jordan, K. Clark, C. R. Herrera. 1982. Ecosystem recovery in Amazon caatinga forest after cutting, cutting and burning, and bulldozer treatments. Oikos 38: 313-320.

Uhl, C. 1987. Factors controlling succession following slash-and-burn agriculture in Amazonia. Journal of Ecology 75: 377-407.

Uhl, C., R. Buschbacher, E. A. S. Serrao. 1988. Abandoned pastures in Eastern Amazonia. I. Patterns of plant succession. Journal of Ecology 76: 663-681.

Urbano, A. R., W. S. Keeton. 2017. Carbon dynamics and structural development in recovering secondary forests of the northeastern U.S. Forest Ecology and Management 392: 21-35.

Wang, J. R., A. L. Zhong, S. W. Simard, J. P. Kimmins. 1996. Aboveground biomass and nutrient accumulation in an age sequence of paper birch (*Betula papyrifera*) in the Interior Cedar Hemlock zone, British Columbia. Forest Ecology and Management 83: 27-38.

Williams-Linera, G. 1983. Biomass and nutrient content in two successional stages of tropical wet forest in Uxpanapa, Mexico. Biotropica 15: 275-284.

Yepes, A. P., J. I. del Valle, S. L. Jaramillo, S. A. Orrego. 2010. Recuperación estructural en bosques sucesionales andinos de Porce (Antioquia, Colombia). Rev. Biol. Trop. 58: 427-445.

Zribi, L., Chaar, H., Khaldi, A., Hanchi, B., Mouillot, F., Gharbi, F. 2016. Estimate of biomass and carbon pools in disturbed and undisturbed oak forests in Tunisia. Forest Systems 25: e060.

## 2.3. Agroforestry

Akyeampong, E., L. Hitimana, E. Torquebiau, P. C. Munyemana. 1999. Multistrata agroforestry with beans, bananas and *Grevillea robusta* in the highlands of Burundi. Expl. Agric. 35: 357–369.

Albrecht, A., S. T. Kandji. 2003. Carbon sequestration in tropical agroforestry systems. Agriculture Ecosystems and Environment 99: 15-27.

Alegre, J., L. Arévalo, A. Ricse. 2000. Reservas de carbono y emisión de gases con diferentes sistemas de uso de la tierra en dos sitios de la Amazonía Peruana. CORPOICA; ICRAF; Universidad de Gales. Taller Internacional: Métodos para Investigación en Sistemas Agroforestales. Tibaitatá, Colombia.

Alegre, J. C., L. X. Montoya Vilcahuaman, G. Correa. 2007. Geração da curva alométrica para avaliar as reservas de carbono em plantios de erva-mate, no sul do Brasil. Colombo: Embrapa Florestas. Boletim de pesquisa e desenvolvimento. 19 pp.

Alpizar, L. H. W. Fassbender, J. Heuveldop, H. Folster, G. Henriques. 1986. Modelling agroforestry systems of cacao (*Theobroma cacao*) with laurel (*Cordia alliodora*) and poro (*Erythrina poeppigiana*) in Costa Rica. Agroforestry Systems 4: 175-189.

Amézquita, M. C., M. Ibrahim, P. Buurman, E. Amézquita. 2005. Carbon sequestration in pastures, silvopastoral systems and forests in four regions of the Latin American Tropics. J. Sustain. For. 21: 21-49.

Anderson, E. K., H. Zerriffi. 2012. Seeing the trees for the carbon: agroforestry for development and carbon mitigation. Climatic Change 115: 741-757.

Andrade, H. J., L. M. Marín, D. P. Pachón. 2014. Fijación de carbono en sistemas de producción de café (*Coffea arabica* L.) en el Líbano, Tolima, Colombia. Bioagro 26: 127-132.

Andrade, H. J., R. Brook, M. Ibrahim. 2008. Growth, production and carbon sequestration of silvopastoral systems with native timber species in the dry lowlands of Costa Rica. Plant and Soil 308: 11–22.

Aristizabal, J., A. Guerra. 2002. Estimación de la tasa de fijación de C en el sistema agroforestal Nogal Cafetero (*Cordia alliodora*) – Cacao (*Theobroma cacao* L.) – Plátano (*Musa paradisiaca*). Univ. Distrital Francisco José de Caldas, Fac. Med. Amb. y Rec. Nat., Bogota.

Bayala, J., S. J. Ouedraogo, Z. Teklehaimanot. 2008. Rejuvenating indigenous trees in agroforestry parkland systems for better fruit production using crown pruning. Agroforest. Syst. 72: 187–194.

Bolfe, E. L., M. Batistella, M. C. Ferreira. 2011. Correlação entre o carbono de sistemas agroflorestais e índices de vegetação. Anais XV Simpósio Brasileiro de Sensoriamento Remoto - SBSR, Curitiba, PR, Brasil, pp.1705.

Brancher, T. 2010. Estoque e ciclagem de carbono de sistemas agroflorestais em Tome Azu, Amazonia Oriental. MS Thesis, Univdersidad Nacional do Para, Belem, Brazil. 58 pp.

Budiadi, I. H. T. 2010. Comparison of carbon sequestration between multiple-crop, single-crop and monoculture agroforestry systems of *Melaleuca* in Java, Indonesia. J. Trop. For. Sci. 2: 378–388.

Casanova, L. F., M. J. Caamal, A. J. Petit, S. F. Solorio, C. J. Castillo. 2010. Almacenamiento de carbono en la biomasa de *Leucaena leucocephala* y *Guazuma ulmifolia* asociadas y en monocultivo en Xmatkuil, Yucatán. In: Sixth International Congress of Agroforesttry for Sustainable Cattle Production, Panamá, Panamá. M. Ibrahim and E. Murgueitio (eds). Technical Series no. 15, 160 pp.

Chakeredza, S., L. Hove, F. K. Akinnifesi, S. Franzel, O. C. Ajayi, G. Sileshi. 2007. Managing fodder trees as a solution to human-livestock food conflicts and their contribution to income generation for smallholder farmers in Southern Africa. Natural Resources Forum 31: 286–296.

Dhyani, S. K., D. S. Chauhan, D. Kumar, R. V. Kushwaha, S. T. Lepcha. 1996. Sericulture based agroforestry systems for hilly areas of North-East India. Agroforest. Syst. 34: 1–12.

Dhyani, S. K., K. A. Kareemulla, A. K. Handa. 2009. Agroforestry potential and scope for development across agroclimatic zones in India. Indian J. For. 32: 181–190.

Dixon, R. K., J. K. Winjum, P. E. Schroeder. 1993. Conservation and sequestration of carbon: the potential of forest and agroforest management practices. Global Environmental Change 3: 159–173.

Dixon, R. K., J. K. Winjum, K. J. Andrasko, J. J. Lee, P. E. Schroeder. 1994. Integrated land-use systems: assessment of promising agroforest and alternative land-use practices to enhance carbon conservation and sequestration. Climatic Change 27: 71–92.

Dossa, E. L., E. C. Fernandes, W. S. Reid, K. Ezui. 2008. Above- and belowground biomass, nutrient and carbon stocks contrasting an open grown and a shaded coffee plantation. Agroforest. Syst. 72: 103-115.

Glenday, J. 2008. Carbon storage and emissions offset potential in an African dry forest, the Arabuko-Sokoke Forest, Kenya. Environmental Monitoring and Assessment 142: 85–95.

Gockowski, J., D. Sonwa. 2011. Cacao intensification scenarios and their predicted impact on CO_2_ emissions, biodiversity conservation and rural livelihoods in the Guinea rainforest of West Africa. Environ. Manage. 48: 307–321.

Hauser, S. 2002. Volunteer biomass production between multipurpose tree hedgerows after two years of fallow in southern Cameroon. Agroforest. Syst. 55: 130–147.

Henry, M., P. Tittonell, R. J. Manlay, M. Bernoux, A. Albrecht, B. Vanlauwe. 2009. Biodiversity, carbon stocks and sequestration potential in aboveground biomass in smallholder farming systems of western Kenya. Agri. Ecosyst. Environ. 129: 238–252.

Hergoualch, K., E. Blanchart, U. Skiba, C. Hénault, J. M. Harmand. 2012. Changes in carbon stocks and net balance of greenhouse gases (CO_2_, CH_4_, N_2_O) in a coffee (*Coffea arabica*) monoculture and a coffee agroforestry system with *Inga densiflora* in Costa Rica. Agriculture, Ecosystems and Environment 148: 102-110.

Ibrahim, M., M. Chacón, C. Cuartas, J. Naranjo, G. Ponce, P. Vega, F. Casasola, J. Rojas. 2007. Almacenamiento de carbono en el suelo y la biomasa arbórea en sistemas de usos de la tierra en paisajes ganaderos de Colombia, Costa Rica y Nicaragua. Agroforestería en las Américas 45: 27-36.

Joshi, L., R. Pasha, E. Mulyoutami, H. J. Beukema. 2011. Rubber agroforestry and PES for preservation of biodiversity in Bungo district, Sumatra. In: Payments for ecosystem services and food security. Ottaviani, D., N. E. Scialabba (eds). FAO, Rome, Italy. Pp. 114–135.

Kang, B. 1997. Alley cropping soil productivity and nutrient recycling. For. Ecol. Manage. 91: 75–82.

Kaonga, M. L., T. P. Bayliss-Smith. 2009. Carbon pools in tree biomass and the soil in improved fallows in eastern Zambia. Agroforest. Syst. 76: 37-51.

Kaya, B., P. E. Hildebrand, P. K. R. Nair. 2000. Modeling changes in farming systems with the adoption of improved fallows in southern Mali. Agricultural Systems 66: 51–68.

Kirby, K. R., C. Potvin. 2007. Variation in carbon storage among tree species: Implications for the management of a small-scale carbon sink project. Forest Ecology and Management 246: 208–221.

Kuersten, E., P. Burschel. 1993. CO_2_-mitigation by agroforestry. Water Air and Soil Pollution 70: 533–544.

Kumar, B. M., S. S. Kumar, R. F. Fisher. 1998. Intercropping teak with *Leucaena* increases tree growth and modifies soil characteristics. Agroforest. Syst. 42: 81–89

Kumar, B. M., S. J. George, V. Jamaludheen, T. K. Suresh. 1998. Comparison of biomass production, tree allometry and nutrient use efficiency of multipurpose trees grown in woodlot and silvopastoral experiments in Kerala, India. For. Ecol. Manage. 112: 145–163.

Kumar, B. M. 2006. Carbon sequestration potential of tropical homegardens. In: Tropical Homegardens: A Time-Tested Example of Sustainable Agroforestry. Kumar, B. M., P. K. R. Nair (eds). Springer Science, The Netherlands, pp 185–204.

Kumar, B. M. 2011. Species richness and aboveground carbon stocks in the homegardens of central Kerala, India. Agric. Ecosyst. Environ. 140: 430–440.

Lasco, R. D., P. D. Suson. 1999. A *Leucaena leucocephala*-based indigenous fallow system in central Philippines: the Naalad system. Int. Tree Crops J. 10: 161–174.

Lin, B. B. 2007. Agroforestry management as an adaptive strategy against potential microclimate extremes in coffee agriculture. Agric. For. Meteorol. 144: 85–94.

Lott, J. E., S. B. Howard, C. K. Ong, C. R. Black. 2000. Long-term productivity of a *Grevillea robusta*-based overstory agroforestry system in semi-arid Kenya: I. Tree growth. For. Ecol. Manage. 139: 175–186.

Makumba, W., F. K. Akinnifesi, B. Janssen, O. Oenema. 2007. Long-term impact of a *gliricidia*-maize intercropping system on carbon sequestration in southern Malawi. Agric. Ecosyst. Environ. 118: 237–243.

Mena, V. E., H. J. Andrade, C. M. Navarro. 2011. Biomasa y carbono almacenado en sistemas agroforestales con café y en bosques secundarios en un gradiente altitudinal en Costa Rica. Agroforesteria Neotropical 1: 1-16.

Messa Arboleda, H. F. 2009. Balance de gases de efecto invernadero en un modelo de producción de ganadería doble propósito con alternativas silvopastoriles en Yaracuy, Venezuela. M.S. Thesis, CATIE, Turrialba, Costa Rica. 225 pp.

Montagnini, F. 2011. Restoration of degraded pastures using agrosilvopastoral systems with native trees in the Neotropics. In: Montagnini, F., W. Francesconi, E. Rossi (eds.). Agroforestry as a tool for landscape restoration. Nova Science Publishers, New York. Pp. 55-68.

Morel, A. C., S. S. Saatchi, Y. Malhi, N. J. Berry, L. Banin, D. Burslem, R. Nilus, R. C. Ong. 2011. Estimating aboveground biomass in forest and oil palm plantation in Sabah, Malaysian Borneo using ALOS PALSAR data. For. Eco. Manage. 262: 1786–1798.

Muthuri, C. W., C. K. Ong, C. R. Black, V. W. Ngumi, B. M. Mati. 2005. Tree and crop productivity in *Grevillea, Alnus* and *Paulownia*-based agroforestry systems in semi-arid Kenya. For. Ecol. Manage. 212: 23–39.

Mutuo, P.C. 2005. Potential of agroforestry for carbon sequestration and mitigation of greenhouse gas emissions from soils in the tropics. Nutrient Cycling in Agroecosystems 71: 43–54.

Nair, P. K. R., B. M. Kumar, V. D. Nair. 2009. Agroforestry as a Strategy for Carbon Sequestration. Journal of Plant Nutrition and Soil Science 172: 10-23.

Nair, P. K. R., V. D. Nair, B. M. Kumar, J. M. Showalter. 2010. Carbon sequestration in agroforestry systems. Adv. Agron. 108: 237–307.

Ollivier, J., C. Daniel, S. Braconnier. 1994. Food crop intercropping with young coconut palms examples in Vanuatu. Oleagineux 49: 91–108.

Parrotta, J. A. 1999. Productivity, nutrient cycling, and succession in single- and mixed-species plantations of *Casuarina equisetifolia*, *Eucalyptus robusta*, and *Leucaena leucocephala* in Puerto Rico. For. Ecol. Manage. 124: 45–77.

Peichl, M., N. Thevathasan, A. Gordon, J. Huss, R. Abohassan. 2006. Carbon sequestration potentials in temperate tree-based intercropping systems. Agroforest. Syst. 66: 243–257.

Poschen, P. 1986. An evaluation of the *Acacia albida*-based agroforestry practices in the Hararghe highlands of Ethiopia. Agroforest. Syst. 4: 129–143.

Roshetko, J. M., M. Delaney, K. Hairiah, P. Purnomosidhi. 2002. Carbon stocks in Indonesian home garden systems: Can smallholder systems be targeted for increased carbon storage? Am. J. Alt. Agri. 17: 138–148.

Schroth, G., S. A. D'Angelo, W. G. Teixeira, D. Haag, R. Lieberei. 2002. Conversion of secondary forest into agroforestry and monoculture plantations in Amazonia: consequences for biomass, litter and soil carbon stocks after 7 years. For. Ecol. Manage. 163: 131–150.

Schroth, G., M. S. S. da Mota, T. Hills, L. Soto-Pinto, I. Wijayanto, C. W. Arief, Y. Zepeda. 2011. Linking carbon, biodiversity and livelihoods near forest margins: the role of agroforestry. In: Carbon Sequestration in Agroforestry: Processes, Policy, and Prospects. Kumar, B. M., P. K. R. Nair (eds). Springer, Berlin, pp. 179–200.

Sharrow, S. H., S. Ismail. 2004. Carbon and nitrogen storage in agroforests, tree plantations, and pastures in Western Oregon, USA. Agroforestry Systems 60: 123-30.

Sileshi, G., F. K. Akinnifesi, O. C. Ajayi, S. Chakeredza, E. N. Chidumayo, P. Matakala. 2007. Contributions of agroforestry to ecosystem services in the Miombo eco-region of eastern and southern Africa. African J. Environ. Sci. Technol. 1: 68–80.

Soto-Pinto, L. A., M. Anzueto, J. Mendoza, G. Jiménez Ferrer, B. de Jong. 2010. Carbon sequestration through agroforestry in indigenous communities of Chiapas, Mexico. Agroforest. Syst. 78: 39–51.

Sudha, P., V. Ramprasad, M. D. V. Nagendra, H. D. Kulkarni, N. H. Ravindranath. 2007. Development of an agroforestry carbon sequestration project in Khammam district, India. Mitig. Adapt. Strat. Glob. Change 12: 1131–1152.

Swamy, S., S. Puri. 2005. Biomass production and C-sequestration of *Gmelina arborea* in plantation and agroforestry system in India. Agrofor. Syst. 64: 181–195.

Tata, H. L., M. van Noordwijk, M. Werger. 2008. Trees and regeneration in rubber agroforests and other forest-derived vegetation in Jambi (Sumatra, Indonesia). J. For. Res. 5: 1–20.

Takimoto, A., P. K. R. Nair, V. D. Nair. 2008. Carbon stock and sequestration potential of traditional and improved agroforestry systems in the West African Sahel. Agric. Ecosyst. Environ. 125: 159–166.

Tobar, D. L., C. Guerra, C. Sepúlveda, R. Ríos, M. Ibrahim. 2010. Sistemas silvopastoriles y almacenamiento de carbono en fincas ganaderas en Guanacaste, Costa Rica. In: Sixth International Congress of Agroforesttry for Sustainable Cattle Production, Panamá, Panamá. M. Ibrahim and E. Murgueitio (eds). Technical Series no.15, 160 pp.

Torres Rivera, J. A., W. Espinoza Domínguez, L. Krishnamurthy, A. Vázquez Alarcón. 2010. Secuestro de carbono en potreros y bosque caducifolio en Huatusco, Veracruz, México. In: Sixth International Congress of Agroforesttry for Sustainable Cattle Production, Panamá, Panamá. M. Ibrahim and E. Murgueitio (eds). Technical Series no.15, 160 pp.

Tschakert, P., O. T. Coomes, C. Potvin. 2007. Indigenous livelihoods, slash-and-burn agriculture, and carbon stocks in Eastern Panama. Ecological Economics 60: 807–820.

Velarde, S. J., J. Ugarte-Guerra, M. R. Tito, J. L. Capella, M. Sandoval, G. Hyman, A. Castro, J. A. Marín, E. Barona. 2010. Reducing Emissions from All Land Uses in Peru. Final National Report. ASB Partnership for the Tropical Forest Margins. Nairobi, Kenya. 142 p.

Williams, J. N., A. D. Hollander, A. T. O’Green, L. A. Thrupp, R. Hanifin, K. Steenwerth, G. McGourty, L. Jackson. 2011. Assessment of carbon in woody plants and soil across a vineyard-woodland landscape. Carbon Balance Manage. 6: 11.

## 2.4. Mangrove Restoration

Alongi, D. M. 2008. Mangrove forests: resilience, protection from tsunamis, and responses to global climate change. Estuar. Coast. Shelf Sci. 76: 1–13.

Alongi, D. 2012. Carbon Sequestration in Mangrove Forests. Carbon Management 3: 313-322.

Chen, G., N. F. Y. Tam, Y. Ye. 2012. Spatial and seasonal variations of atmospheric N_2_O and CO_2_ fluxes from a subtropical mangrove swamp and their relationships with soil characteristics. Soil Biology and Biochemistry 48: 175-181.

Christensen, B. 1978. Biomass and primary production of *Rhizophora apiculata* Bl. in a mangrove in southern Thailand. Aquatic Botany 4: 43-52.

Golley, F. B., H. T. Odum, R. F. Wilson. 1962 The structure and metabolism of a Puerto Rican red mangrove forest in May. Ecology 43: 9-19.

Kairo, J. G., J. Bosire, J. Langat, B. Kirui, and N. Koedam. 2009. Allometry and Biomass Distribution in Replanted Mangrove Plantations at Gazi Bay, Kenya. In Aquatic Conservation 19: S63-S69.

Kathiresan, K., R. Anburaj, V. Gomathi, K. Saravanakumar. 2013. Carbon Sequestration potential of *Rhizophora mucronate* and *Avicennia marina* as influenced by age, season, growth and sediment characteristics in southeast coast of India. Coastal Conservation Planning and Management 17: 397-408.

Liao, W. B., D. Z. Zheng, S. F. Zeng. 1990. Study on the biomass of *Sonneratia carseolaria* community. J. For. Res. 3: 47–54.

Liao, W. B., D. Z. Zheng, Y. D. Li. 1999. Above ground biomass and nutrient accumulation and distribution in different type *Sonneratia carseolaria- Kandelia candel* mangrove plantations. Chinese J. of Appl. Ecol. 10: 11–15.

Lin, P. 1999. Mangrove ecosystem in China. Science Press, Beijing, pp. 11–250.

Lovelock, C. E., B. K. Sorrell, N. Hancock, Q. Hua, A. Swales. 2010. Mangrove forest and soil development on a rapidly accreting shore in New Zealand. Ecosystems 13: 437-451.

Lugo, A. E. 1992. Comparison of tropical tree plantations with Secondary forests of similar age ecological monographs 62: 1–41.

Matsui, N., Putth, S., and Keiyo, M. 2012. Mangrove Rehabilitation on Highly Eroded Coastal Shorelines at Samut Sakhon, Thailand. Ecology.

Miao, S. Y., G. Z. Chen, Z. T. Chen. 1998. Biomasses and distribution patterns of mangrove populations in Zhanjiang Nature Reserve, Guangdong, China. Guihaia 18: 16–19.

Ong, J. E., W. K. Gong, C. H. Wong. 1981. Ecological monitoring of the Sungai Merbok estuarine mangrove ecosystem. School of Biological Sciences, University Sains Malaysia. Penang, Malaysia. 49 p.

Ong, J. E., W. K. Gong, B. F. Clough. 1995. Structure and productivity of a 20-year-old stand of *Rhizophora apiculata* Bl. mangrove forest. J. Biogeog. 22: 417–24.

Putz, F. E., H. T. Chan. 1986. Tree growth, dynamics, and productivity in a mature mangrove forest in Malaysia. Forest Ecology and Management 17: 211-230.

Ren, H., H. Chen, Z. Li, W. Han. 2010. Biomass accumulation and carbon storage of four different aged *Sonneratia apetala* plantations in Southern China. Plant Soil 327: 279-291.

Ross, M. S., P. L. Ruiz, G. J. Telesnicki, J. F. Meeder. 2001. Estimating aboveground biomass and production in mangrove communities of Biscayne National Park, Florida (USA). Wetlands Ecol. Manage. 9: 27–37.

Salmo III, S. G., N. C. Duke. 2010. Establishing mollusk colonization and assemblage patterns in planted mangrove stands of different ages in Lingayen Gulf, Philippines. Wetlands Ecol. Manage. 18: 745–754.

Salmo III, S. G., C. Lovelock, N. C. Duke. 2013. Vegetation and soil characteristics as indicators of restoration trajectories in restored mangroves. Hydrobiologia 720: 1-18.

Sherman, R. E., T. J. Fahey, P. Martinez. 2003. Spatial patterns of biomass and aboveground net primary productivity in a mangrove ecosystem in the Dominican Republic. Ecosystems 6: 384-398.

Sukardjo, S., I. Yamada. 1992. Biomass and productivity of a *Rhizophora mucronata* Lamarck plantation in Tritih, Central Java, Indonesia. For. Ecol. Manage. 49: 195–209.

Warner, J. H. 1990. Successional patterns in a mangrove forest in southwest Florida, USA. M.S. Thesis, University of Southwestern Louisiana, Lafayette, LA. 74 pp.

Yin, Y., H. Q. Fan, X. J. Su. 1993. Study on the biomass of *Avicennia marina* community. Journal of Guangxi Academy of Sciences 9: 19–24.

Zan, Q. J., Y. J. Wang, W. B. Liao. 2001. Biomass and net productivity of *Sonneratia carseolaria-Sonneratia apetala* mangrove forest. Journal of Wuhan Botanical Research 15: 391–397.
