# Supplementary material for: Global carbon dioxide removal rates from forest landscape restoration activities
Source: Carbon Balance Manag. 2018 Nov 20;13:22. doi: 10.1186/s13021-018-0110-8 (PMC6246754; doi:10.1186/s13021-018-0110-8)
Supplement: Supplementary file 2 — Additional file 2. FLR growth curves [file 13021_2018_110_MOESM2_ESM.pptx]

## Slide 1
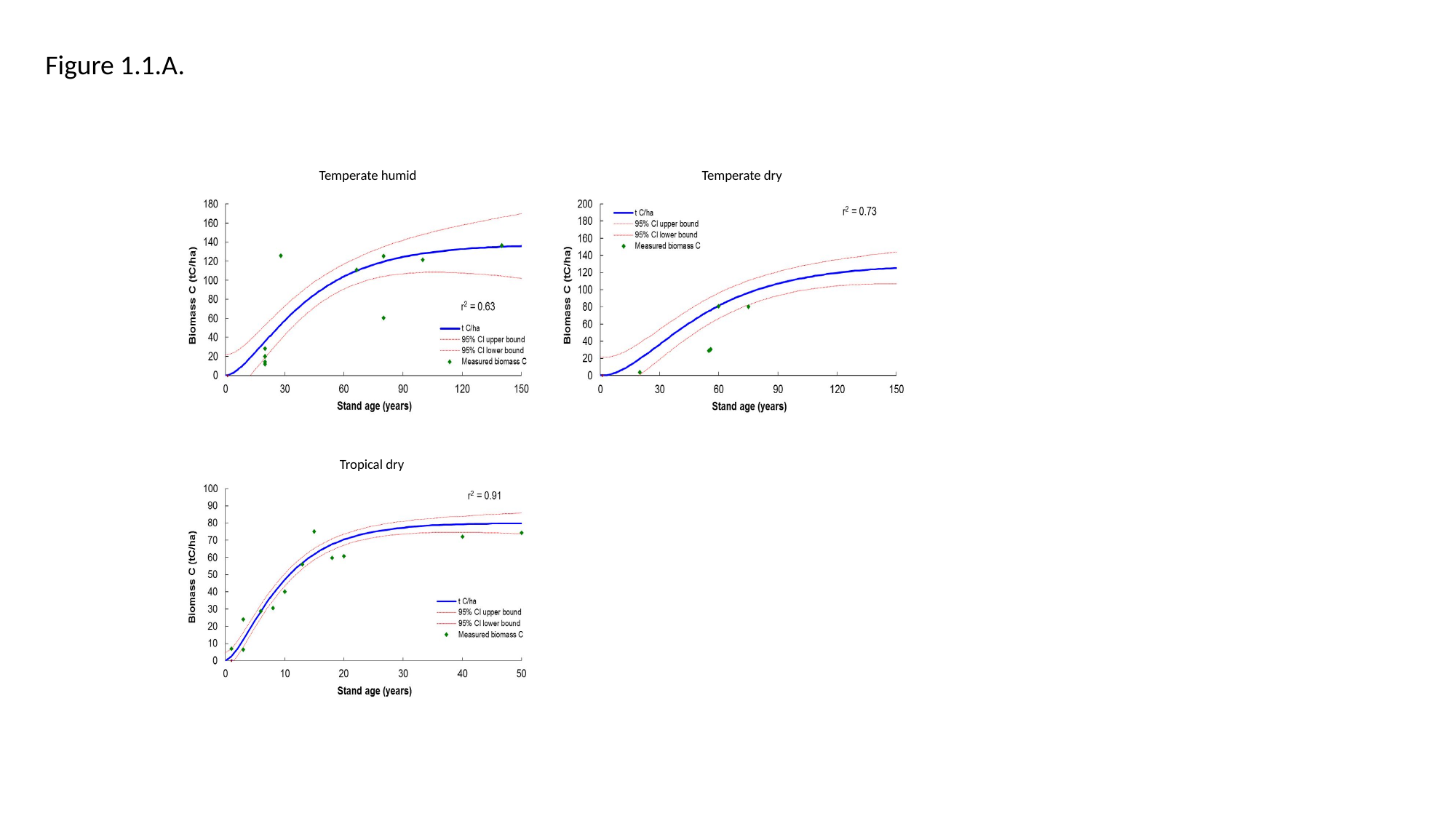

Figure 1.1.A.
Temperate humid
Temperate dry
Tropical dry

## Slide 2
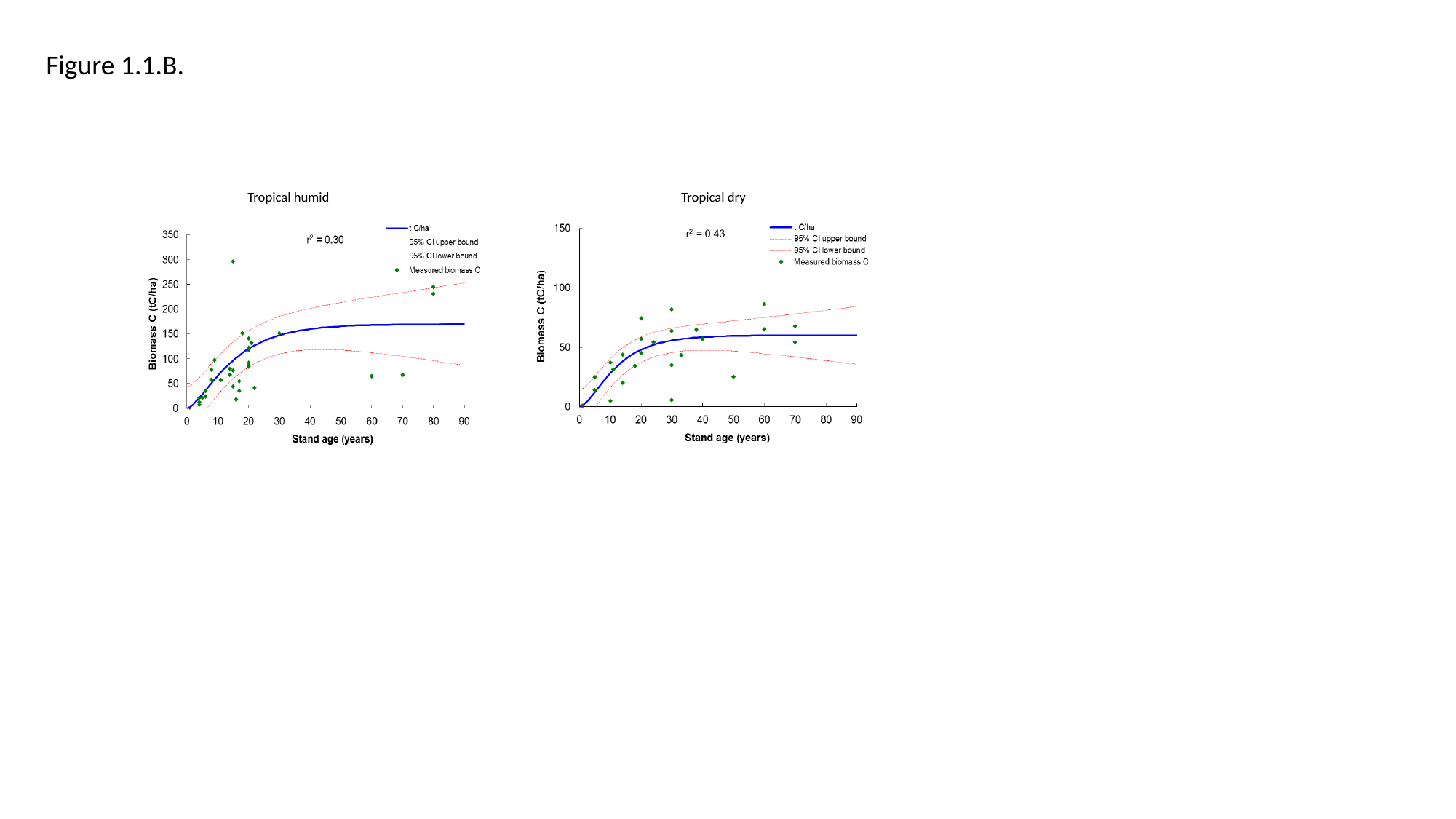

Figure 1.1.B.
Tropical dry
Tropical humid

## Slide 3
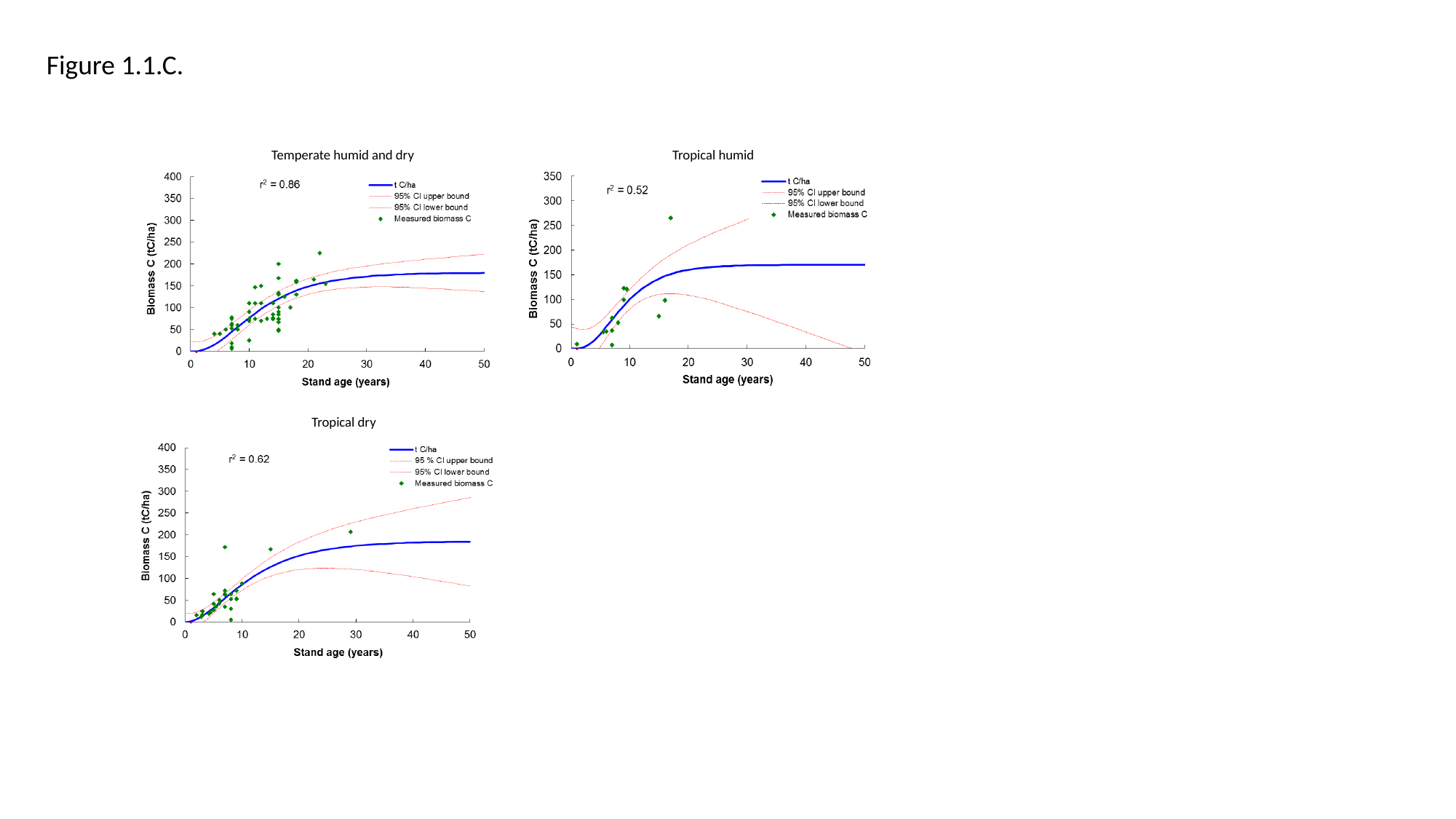

Figure 1.1.C.
Tropical humid
Temperate humid and dry
Tropical dry

## Slide 4
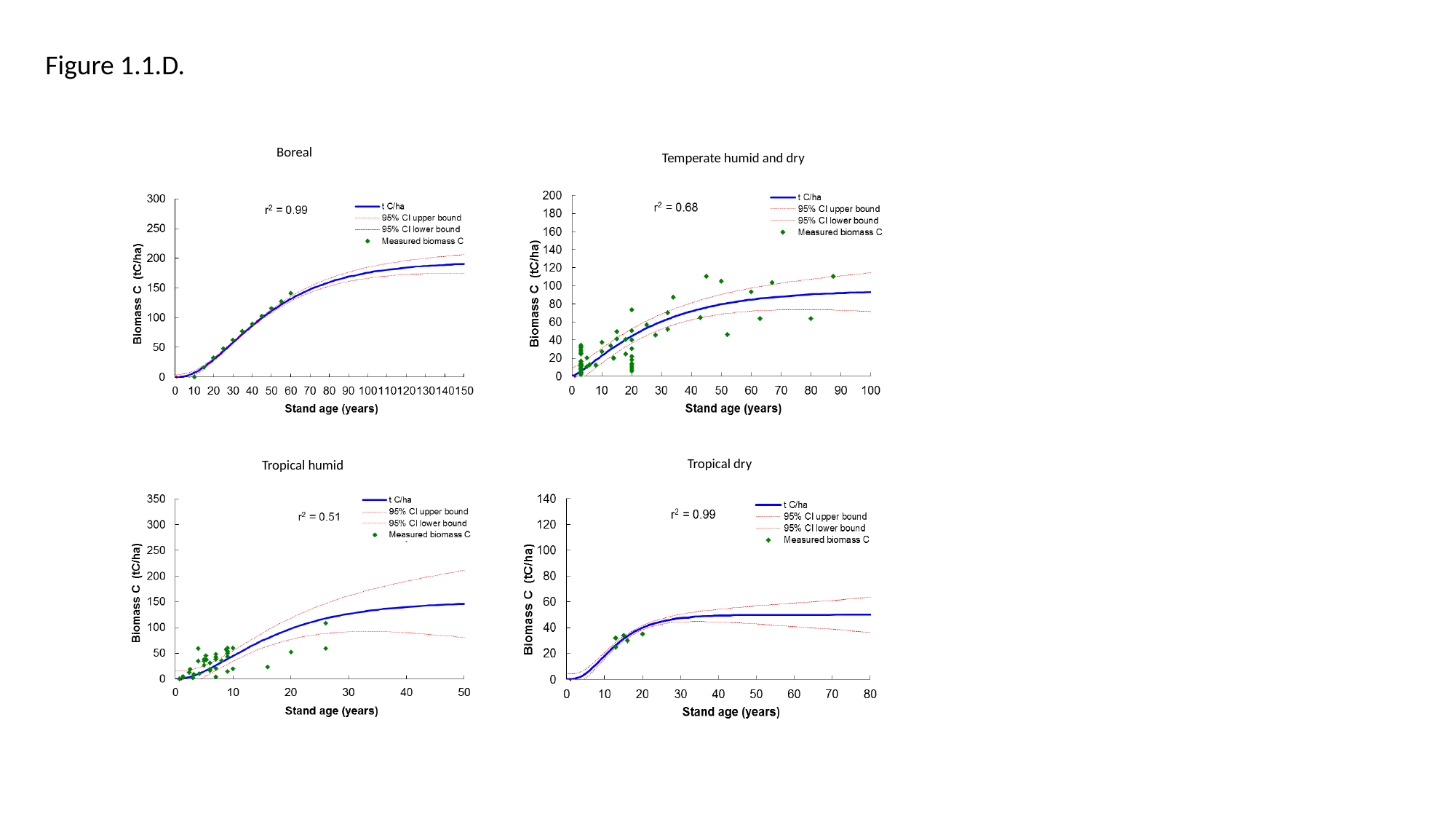

Figure 1.1.D.
Boreal
Tropical dry
Tropical humid
Temperate humid and dry

## Slide 5
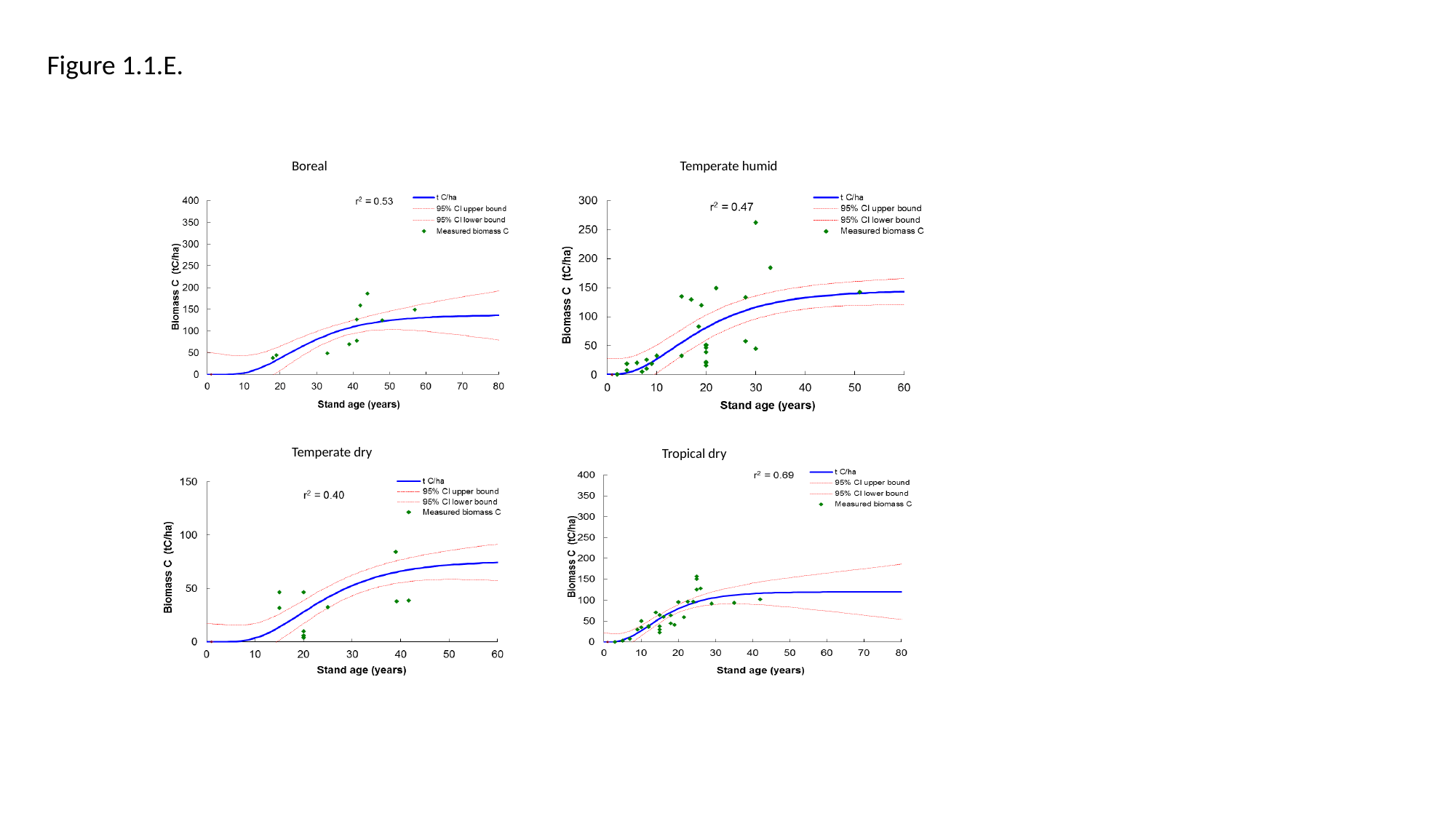

Figure 1.1.E.
Boreal
Temperate humid
Temperate dry
Tropical dry

## Slide 6
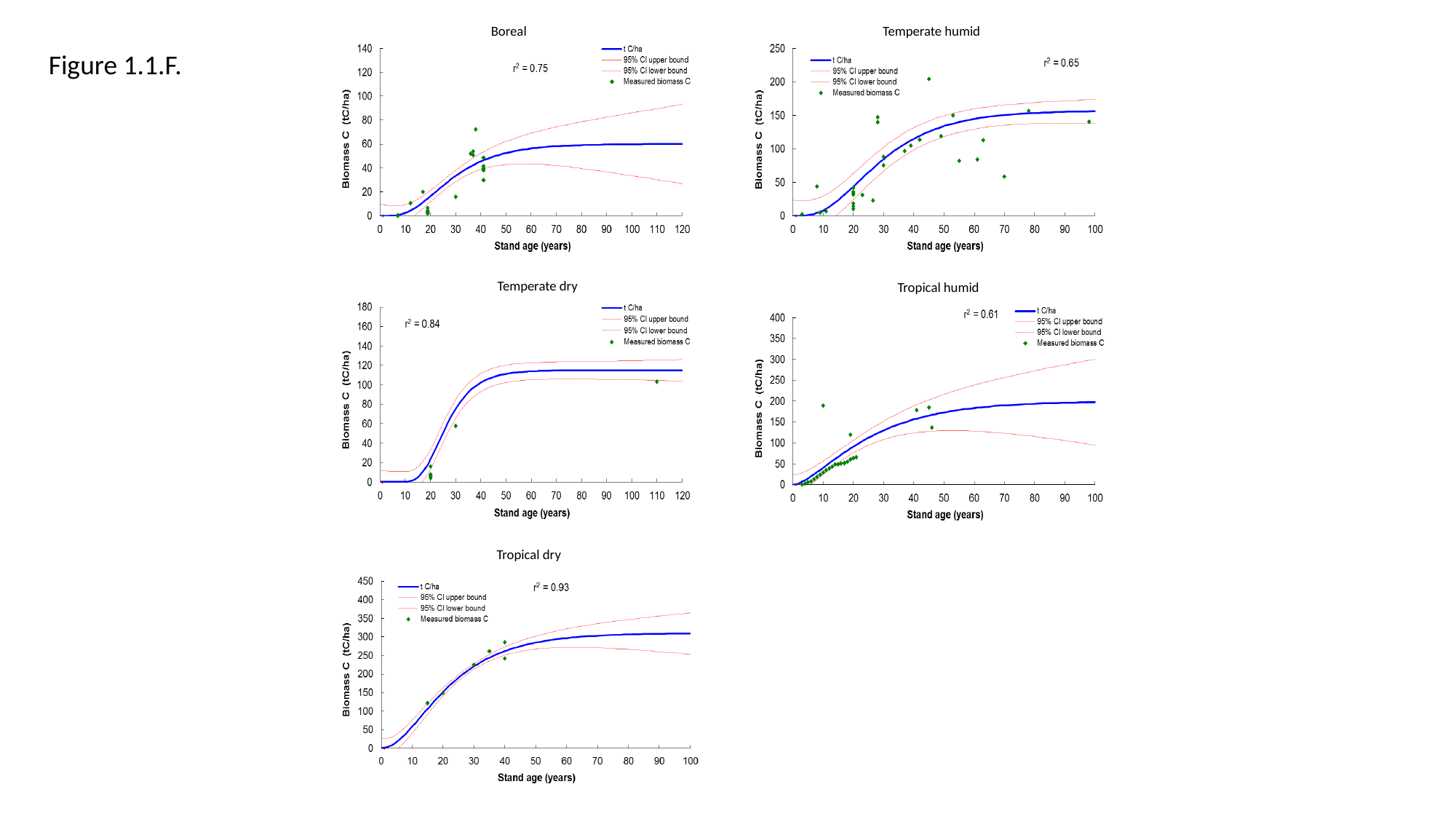

Boreal
Temperate humid
Temperate dry
Tropical humid
Tropical dry
Figure 1.1.F.

## Slide 7
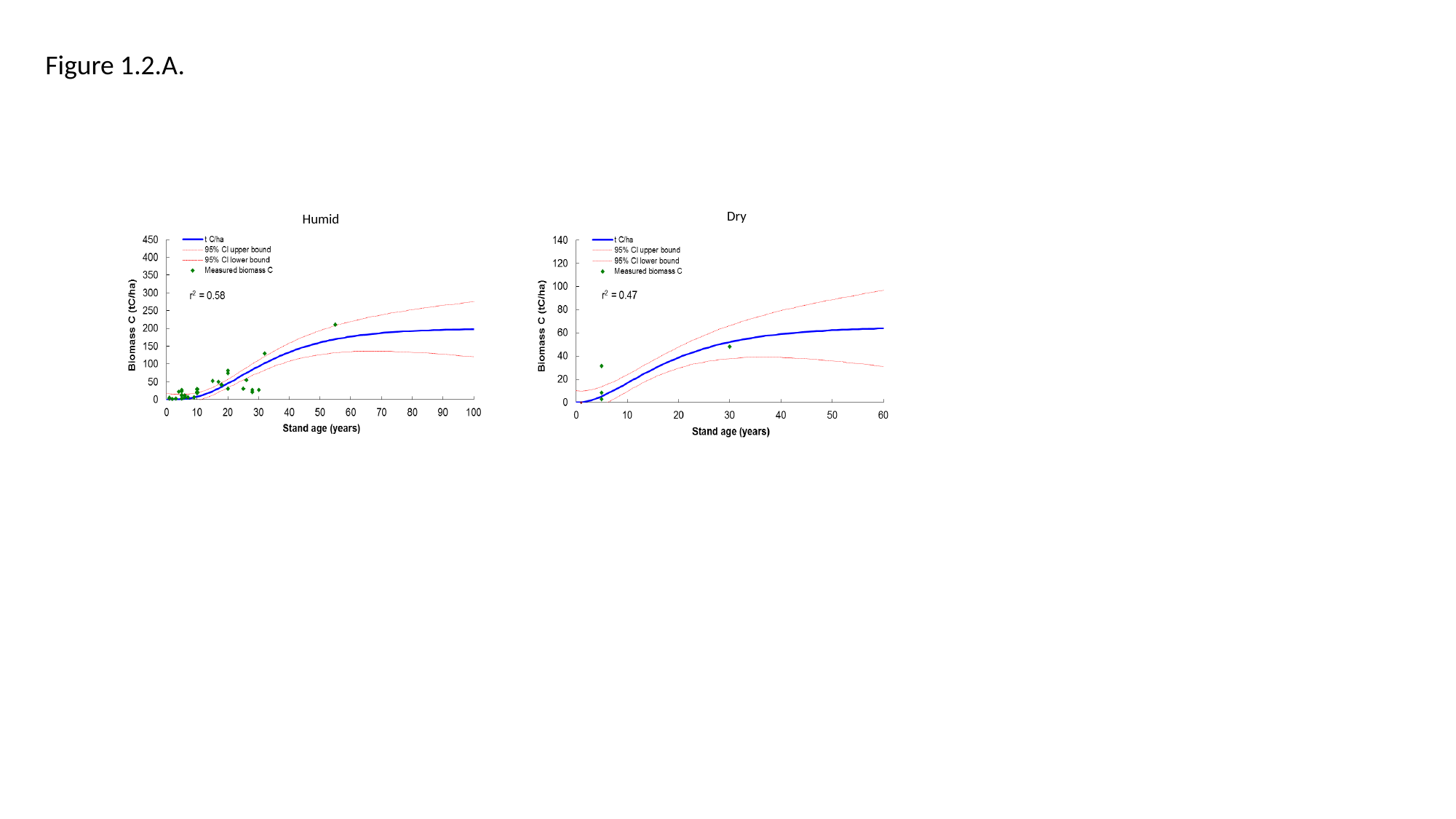

Figure 1.2.A.
Dry
Humid

## Slide 8
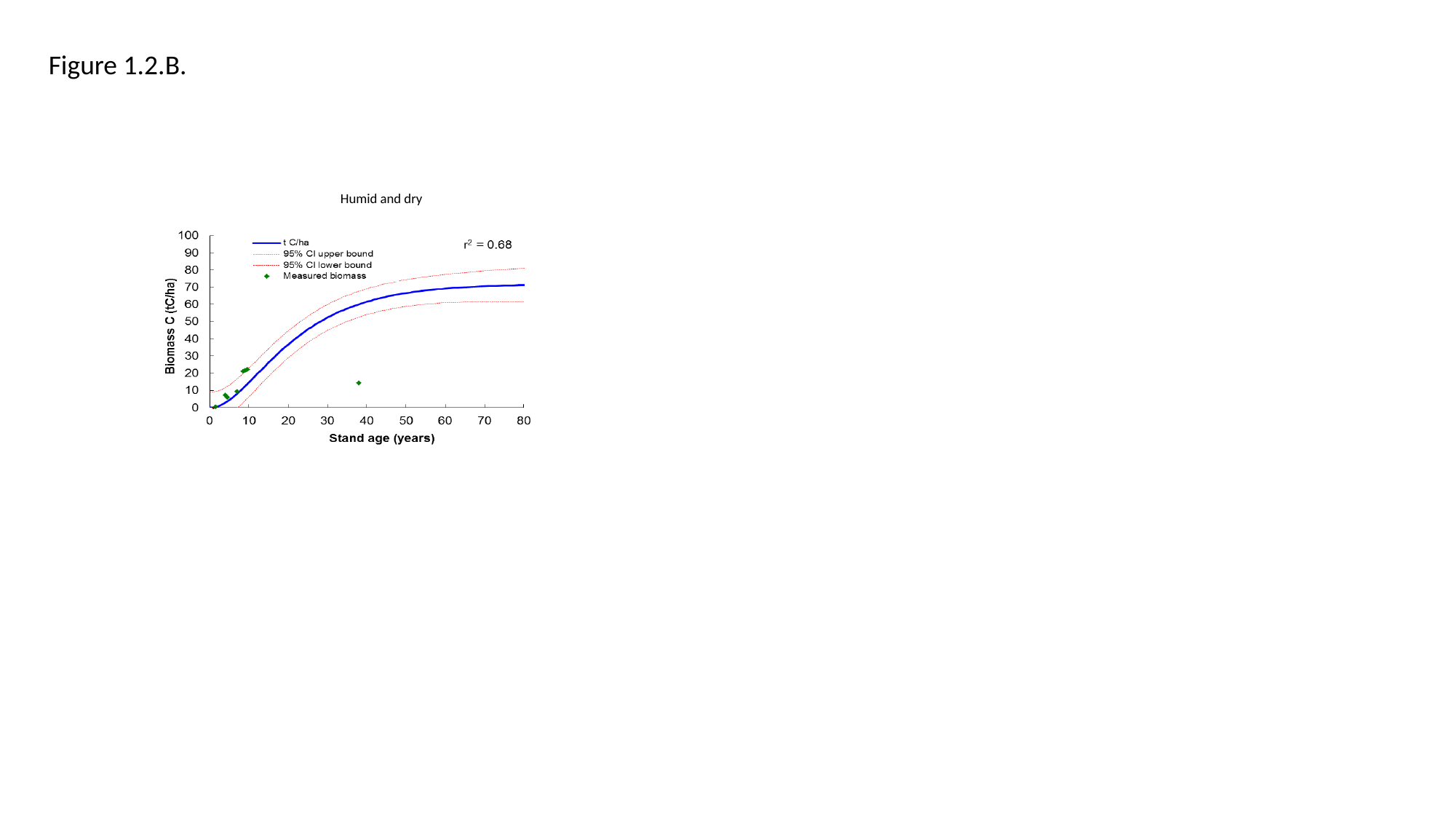

Figure 1.2.B.
Humid and dry

## Slide 9
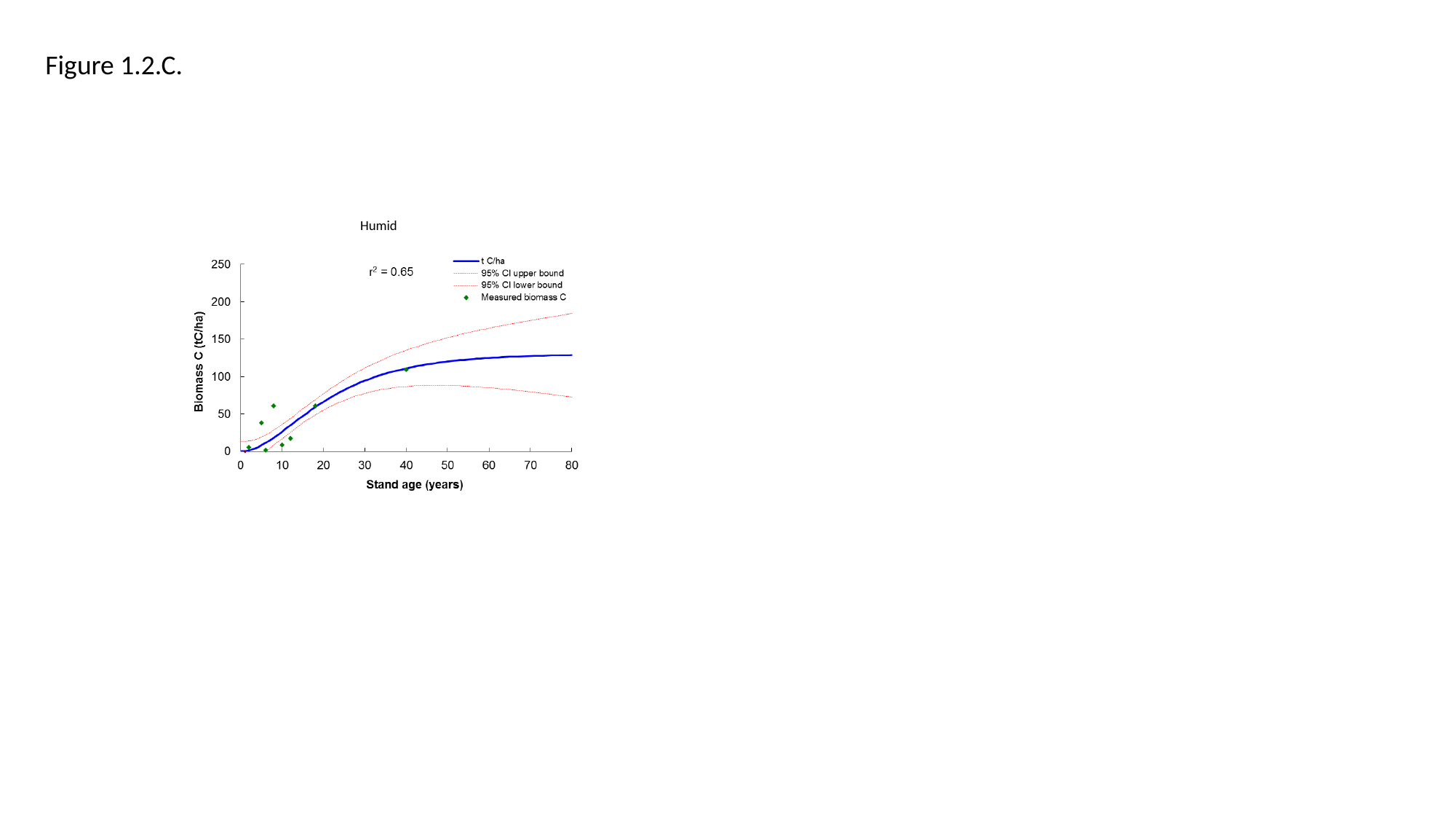

Figure 1.2.C.
Humid

## Slide 10
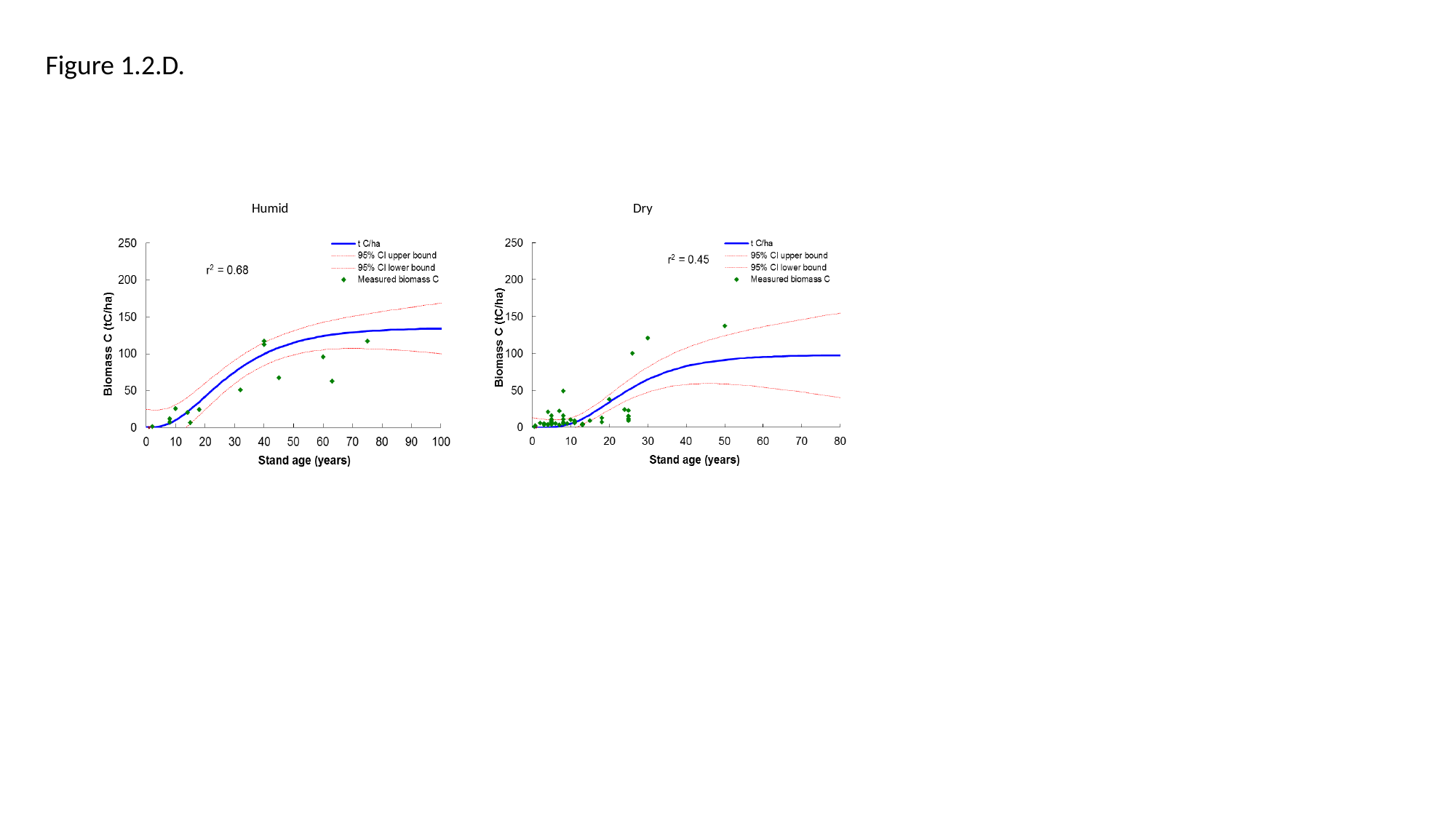

Figure 1.2.D.
Humid
Dry

## Slide 11
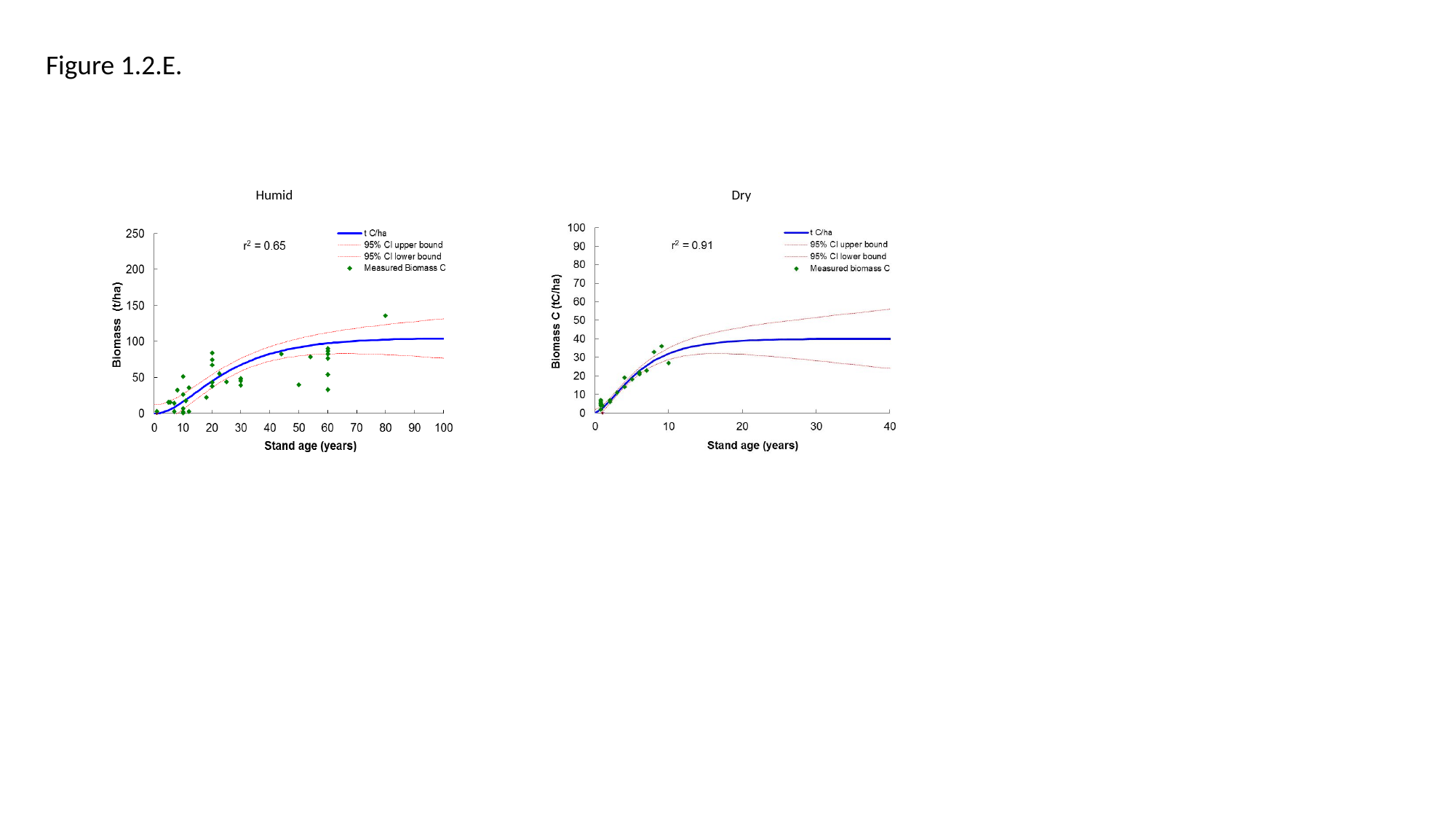

Figure 1.2.E.
Humid
Dry

## Slide 12
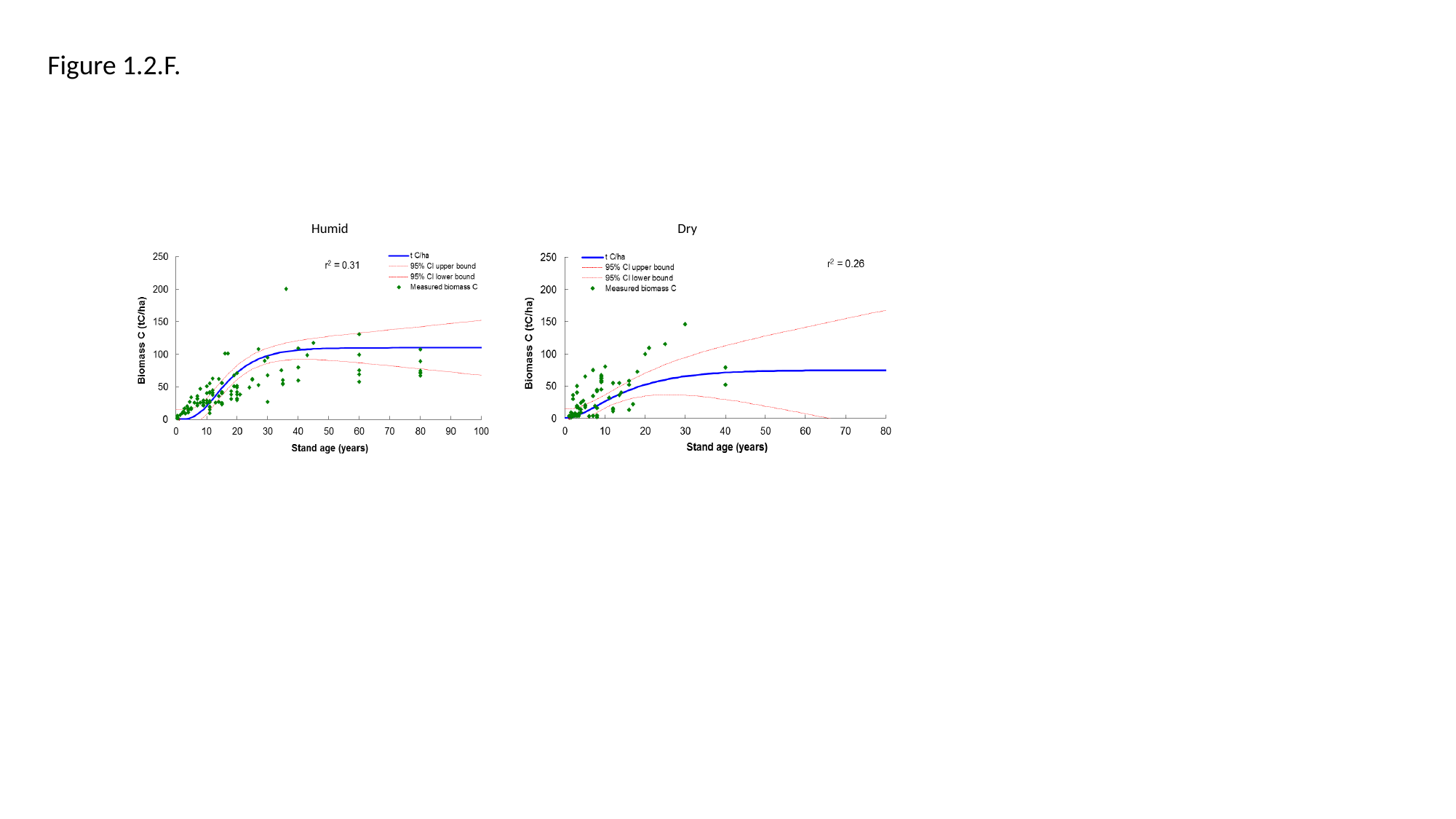

Figure 1.2.F.
Dry
Humid

## Slide 13
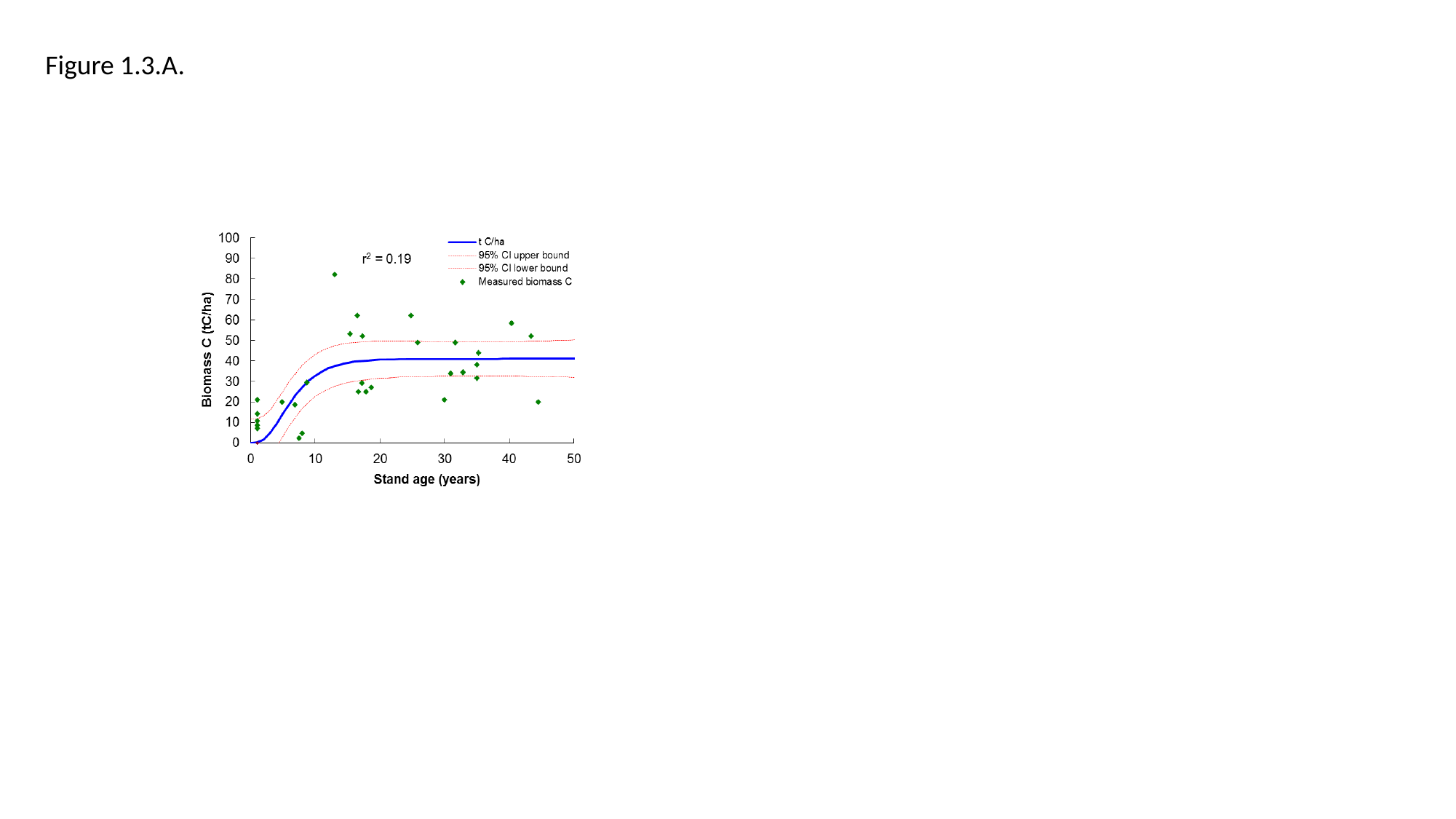

Figure 1.3.A.

## Slide 14
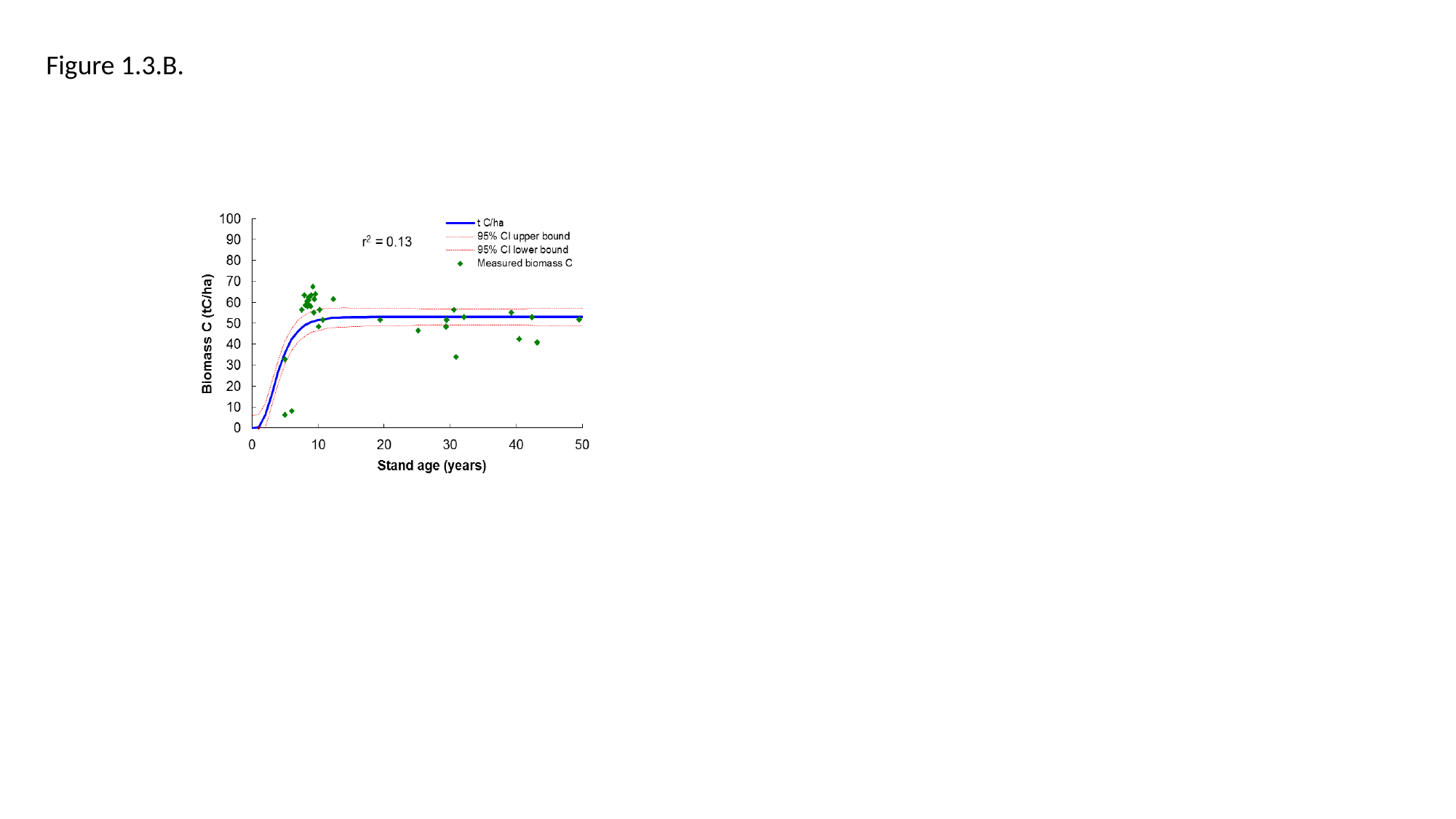

Figure 1.3.B.

## Slide 15
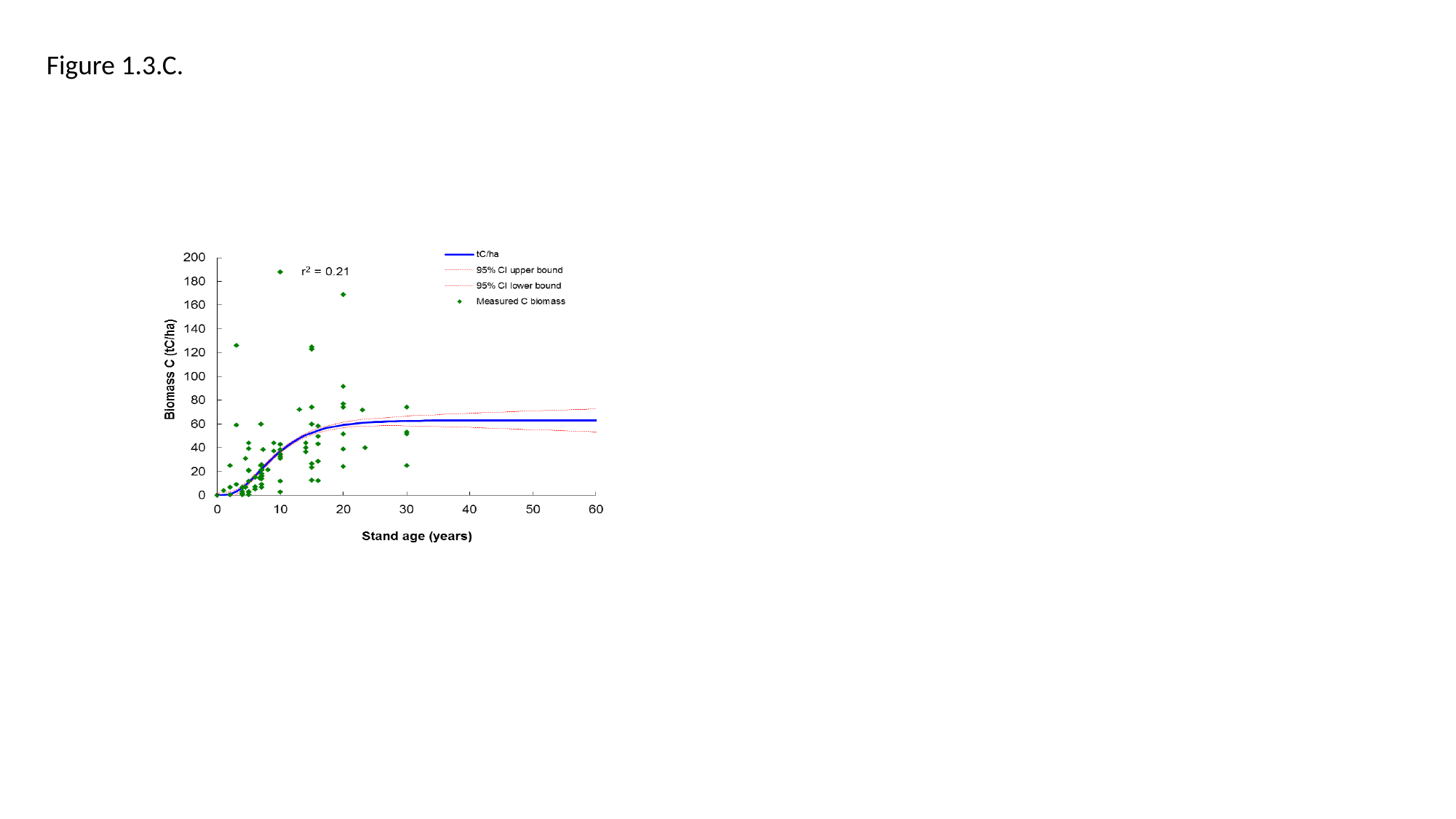

Figure 1.3.C.

## Slide 16
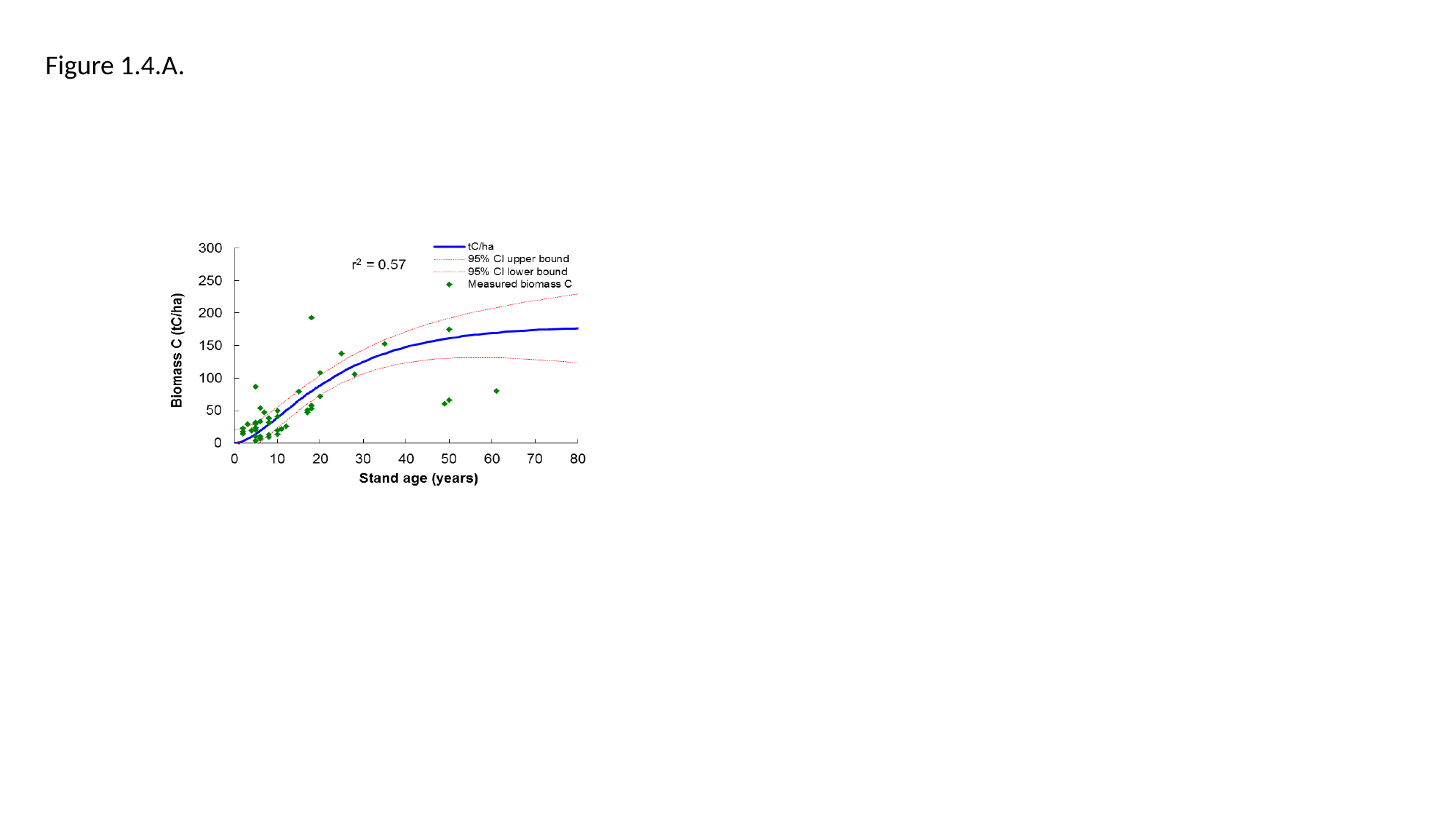

Figure 1.4.A.

## Slide 17
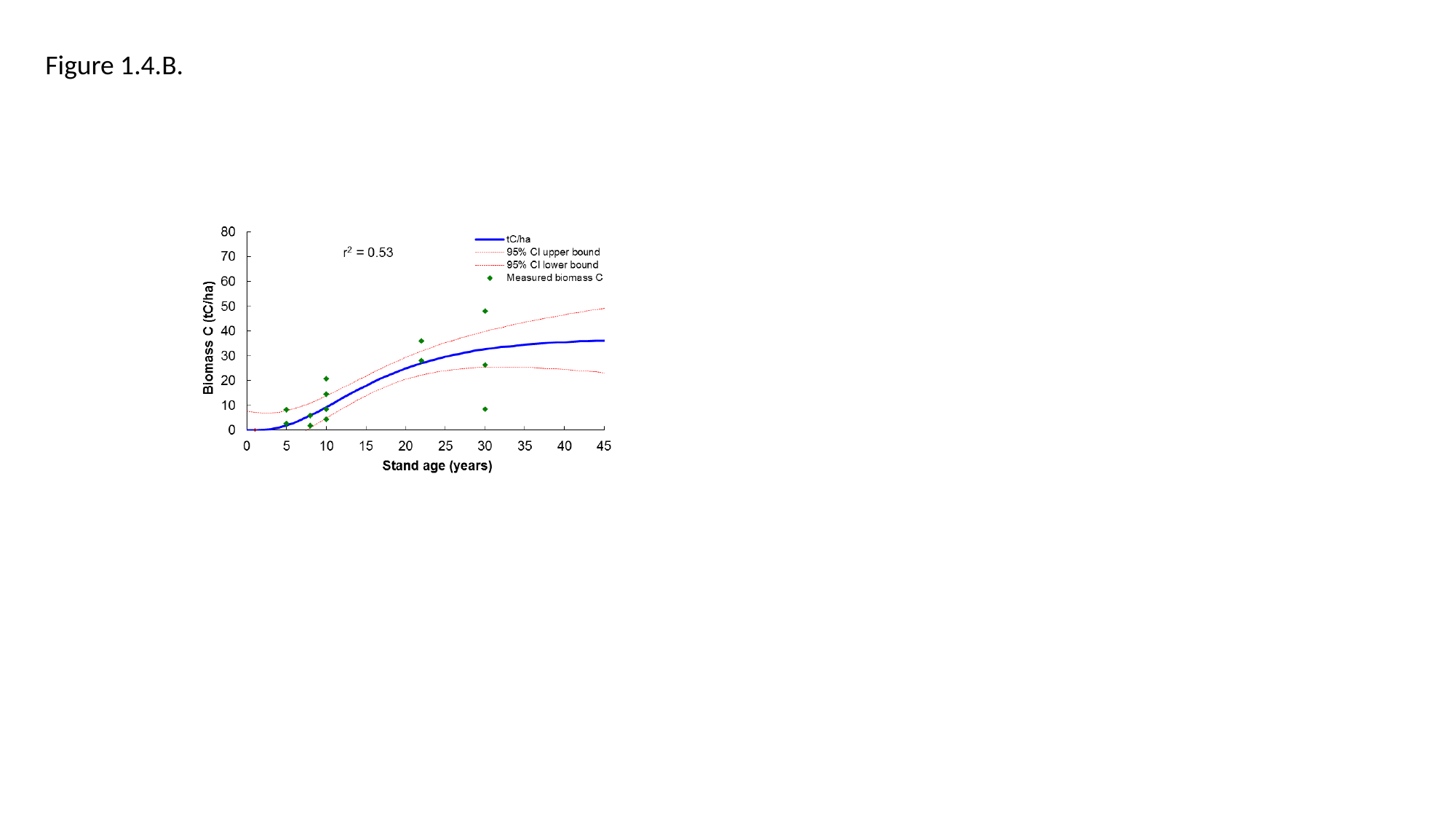

Figure 1.4.B.
